# Circulating 25-hydroxyvitamin D and survival outcomes of colorectal cancer: evidence from population-based prospective cohorts and Mendelian randomisation

**DOI:** 10.1038/s41416-024-02643-5

**Published:** 2024-03-13

**Authors:** Xiaomeng Zhang, Yazhou He, Xue Li, Rasha Shraim, Wei Xu, Lijuan Wang, Susan M. Farrington, Harry Campbell, Maria Timofeeva, Lina Zgaga, Peter Vaughan-Shaw, Evropi Theodoratou, Malcolm G. Dunlop

**Affiliations:** 1 Centre for Global Health, Usher Institute, University of Edinburgh, Edinburgh, UK; 2 Department of Oncology, West China School of Public Health and West China Fourth Hospital, Sichuan University, Chengdu, China; 3 School of Public Health and the Second Affiliated Hospital, Zhejiang University, Hangzhou, China; 4 Department of Public Health and Primary Care, Institute of Population Health, Trinity College Dublin, Dublin, Republic of Ireland; 5 Cancer Research UK Edinburgh Centre, Institute of Genetics and Cancer, University of Edinburgh, Edinburgh, UK; 6 Danish Institute for Advanced Study (DIAS), Department of Public Health, University of Southern Denmark, Odense, Denmark

**Keywords:** Colorectal cancer, Cancer epidemiology

## Abstract

**Background:**

To investigate the association between circulating 25-hydroxyvitamin D (25-OHD) and colorectal cancer (CRC) survival outcomes.

**Methods:**

We conducted analyses among the Study of Colorectal Cancer in Scotland (SOCCS) and the UK Biobank (UKBB). Both cancer-specific survival (CSS) and overall survival (OS) outcomes were examined. The 25-OHD levels were categorised into three groups, and multi-variable Cox-proportional hazard models were applied to estimate hazard ratios (HRs). We performed individual-level Mendelian randomisation (MR) through the generated polygenic risk scores (PRS) of 25-OHD and summary-level MR using the inverse-variance weighted (IVW) method.

**Results:**

We observed significantly poorer CSS (HR = 0.65,95%CI = 0.55–0.76,*P* = 1.03 × 10^−7^) and OS (HR = 0.66,95%CI = 0.58–0.75,*P* = 8.15 × 10^−11^) in patients with the lowest compared to those with the highest 25-OHD after adjusting for covariates. These associations remained across patients with varied tumour sites and stages. However, we found no significant association between 25-OHD PRS and either CSS (HR = 0.98,95%CI = 0.80–1.19,*P* = 0.83) or OS (HR = 1.07,95%CI = 0.91–1.25,*P* = 0.42). Furthermore, we found no evidence for causal effects by conducting summary-level MR analysis for either CSS (IVW:HR = 1.04,95%CI = 0.85–1.28,*P* = 0.70) or OS (IVW:HR = 1.10,95%CI = 0.93–1.31,*P* = 0.25).

**Conclusion:**

This study supports the observed association between lower circulating 25-OHD and poorer survival outcomes for CRC patients. Whilst the genotype-specific association between better outcomes and higher 25-OHD is intriguing, we found no support for causality using MR approaches.

## Introduction

Epidemiological evidence supports an association between 25-hydroxyvitamin D (25-OHD) and CRC risk [1], but not causality [1, 2]. There is very little work has addressed any potential association between 25-OHD and CRC survival, especially in assessing causality. Previous observational evidence yielded inconsistent findings for the association between 25-OHD concentration and CRC survival [3, 4, 5, 6]. These studies were mostly small in sample size (*N* < 1000) and meta-analyses revealed significant between-study heterogeneity [3]. The uncertainty in the evidence could, in part, be attributed to the influence of confounding factors, such as anatomical subsites [7, 8].

In addition to observational evidence, the latest SUNSHINE trial failed to establish causality by detecting a marginal, yet non-significantly improved median progression-free survival among stage IV patients with high-dose versus standard-dose supplementation (8000 IU/d vs 400 IU/d) of vitamin D3 [9]. Another trial among elders from the Finnish population suggested vitamin D3 supplementation (1600 IU/d or 3200 IU/d) did not reduce either CRC incidence or mortality [10]. However, previous meta-analyses demonstrated a clinically meaningful beneficial effect of vitamin D supplementation on CRC survival [11, 12].

Mendelian randomisation study (MR) is an instrumental variable method, which can avoid the influence of confounders, and provide new insights into the potential causal association. The latest genome-wide association study (GWAS) has contributed a wealth of genetic risk variants for 25-OHD and enabled the generation of polygenic risk score (PRS) which has been established as an informative prediction measure of heritable traits [13]. Therefore, in the current study, we first conducted observational studies in the Study of Colorectal Cancer in Scotland (SOCCS) and the UK Biobank (UKBB) controlling for potential confounding effects. We then conducted both individual- and summary-level MR analyses to identify any causality.

## Methods

The SOCCS is a prospective, population-based case-control study aiming to investigate genetic and environmental factors associated with the risk and survival of CRC cases across Scotland. The UKBB is a large-scale prospective cohort with in-depth genetic and health information from the general population of the UK. The circulating 25-OHD level was May-standardised [4, 14] and the rank-based inverse-normal transformation was applied to normalise the distribution of May-standardised 25-OHD levels. Further descriptions of the two cohorts and the measurement of 25-OHD concentration can be found in supplementary methods and in our previous publications [15, 16]. We have considered two up-to-date GWASs for our genetic instruments [17, 18]. Consequently, a total of 133 genetic variants associated with circulating 25-OHD concentration at *p* < 5 × 10^−8^ were selected from the GWAS with 417,580 Europeans from the UKBB and these variants could explain from 5.7% to 10.5% of the variance for the 25-OHD level [17]. At last, 113 variants in UKBB and 107 variants in SOCCS were retained to generate the 25-OHD genetic instrument (Fig. S1). We generated a PRS by adding the weighted dosages of risk alleles for each of the variants. The list of genetic variants for 25-OHD is presented in Tables S1 & S2.

### Statistical analyses

We plotted the predicted hazard ratio of CRC by 25-OHD and no disparity from linearity was observed (Fig. S2). To reduce survivor bias, we restricted the observational analysis to incident cases in UKBB (Fig. S3). Finally, 2936 and 3181 CRC patients who have both 25-OHD levels and survival records from SOCCS and UKBB, respectively, were included in the observational analysis. We categorised 25-OHD levels into three groups using clinical cutoffs defined by the Institute of Medicine [19] (Group 1: <25 nmol/L, Group 2: 25–50 nmol/L and Group 3: >50 nmol/L). We then analysed the associations between circulating 25-OHD and other covariates by one-way analysis of variance for continuous variables or *χ*^2^ test for categorical variables. Survival estimates for patients in each vitamin D group were estimated using the Kaplan-Meier approach. To account for the effects of covariates, we fitted three multivariable Cox proportional hazard models to estimate hazard ratios (HRs) of 25-OHD concentrations taking the group with the lowest 25-OHD level as a reference. The proportional hazards assumption was evaluated using Schoenfeld residuals based on model 3 including all covariates [20]. When any deviations were found, we re-estimated the HRs after stratifying for this covariate. We also investigated the potential gene-environment interaction between 25-OHD and the status of vitamin D receptor (VDR) polymorphism rs11568820 as suggested by previous findings [5]. In addition, we conducted stratified analysis by tumour site and stages based on the data availability.

We performed MR analyses in 5675 and 5847 CRC patients for whom genotype data and survival records were available in SOCCS and UKBB, respectively. An F-statistic less than ten indicates the presence of weak instrument effects [21]. The statistical power was estimated using a non-centrality parameter approach [22]. To perform individual-level MR, the association between PRS and CRC survival was assessed by proportional hazards models, adjusting for age and sex. AJCC stage was adjusted only in SOCCS (unavailable in the UKBB). Whereas for summary-level MR, the same Cox models were fitted to estimate the individual effect of each genetic variant on survival outcomes, after which the overall causal effect was estimated primarily by using the inverse variance-weighted (IVW) method [23]. Given that the MR methods rely on the fulfilment of specific assumptions for each method, we conducted the weighted median-based MR [24], MR-PRESSO [25], MR-RAPS [26] and MR contamination mixture [27] as sensitivity analyses, to enhance the robustness of our estimation [28]. Significant heterogeneity was indicated when Cochran’s Q statistic *P* < 0.10 [29]. We explored potential biases introduced by genetic pleiotropy using the MR-Egger method [30]. We conducted a sensitivity analysis using a PRS generated by the six 25-OHD genetic instruments (PRS_6_) detected by the initial SUNLIGHT GWAS [31]. These six variants were in vitamin D pathway-related genes such as *GC*, *CYP2R1,* and *DHCR7/NADSYN1* and were replicated by the other GWASs.

Fixed-effect meta-analysis was performed to pool the estimates from the two datasets. The significance threshold for main analyses for both observational and MR studies was set at 0.05 (two-sided). We applied a Bonferroni corrected significance threshold (*P* < 0.004) to correct false positive rates from multiple tests in the subgroup analyses. All statistical analyses were performed on R v3.6.3. MR analyses were performed using the package ‘MendelianRandomization’ [32], ‘MRPRESSO’ [25], and ‘mr.raps’ [26].

## Results

### Observational studies

A total of 2936 and 3181 CRC patients with 20,336 and 19,675 total person-years of follow-up from the SOCCS and UKBB respectively were included in this study. The basic characteristics of CRC patients are presented in Table 1. At the time of sampling, 29.56% and 12.54% of patients fulfilled the criteria for vitamin D deficiency (<25 nmol/L), and 47.10% and 47.72% were at insufficient levels (25–50 nmol/L) for SOCCS and UKBB respectively. Distributions of covariates for each 25-OHD tertile can be found in Tables S3 and S4.

**Table 1:** Summarised characteristics of colorectal cancer patients.

	SOCCS	UKBB
CRC patients	2936	3181
25-OHD (nmol/L)	40.01 (24.26)	46.46 (19.85)
<25 nmol/L	29.56%	12.54%
25–50 nmol/L	47.10%	47.72%
>50 nmol/L	23.34%	39.74%
Age of diagnosis (years)	66.15 (9.87)	65.28 (6.63)
Proportion of females	1239 (42.20%)	1309 (41.15%)
BMI	26.69 (4.28)	28.01 (4.62)
Follow-up years	6.93 (4.89)	6.19 (3.46)
AJCC stages		
I	547 (18.63%)	/
II	1015 (34.57%)	/
III	1056 (35.97%)	/
IV	318 (10.83%)	/
Season of blood collection		
Autumn	794 (27.04%)	745 (23.42%)
Spring	758 (25.82%)	892 (28.04%)
Summer	665 (22.65%)	865 (27.19%)
Winter	719 (24.49%)	679 (21.35%)
Cause of death		
CRC	684 (14.56%)	793 (24.93%)
All	1013 (31.39%)	1164 (36.59%)

The continuous variables were described by using the mean and the corresponding standard deviation; the categorical variables were described by using numbers and proportions.

*SOCCS* Study of Colorectal Cancer in Scotland, *UKBB* UK Biobank, *SD* standard deviation, *BMI* body mass index.

**Fig. 1:**
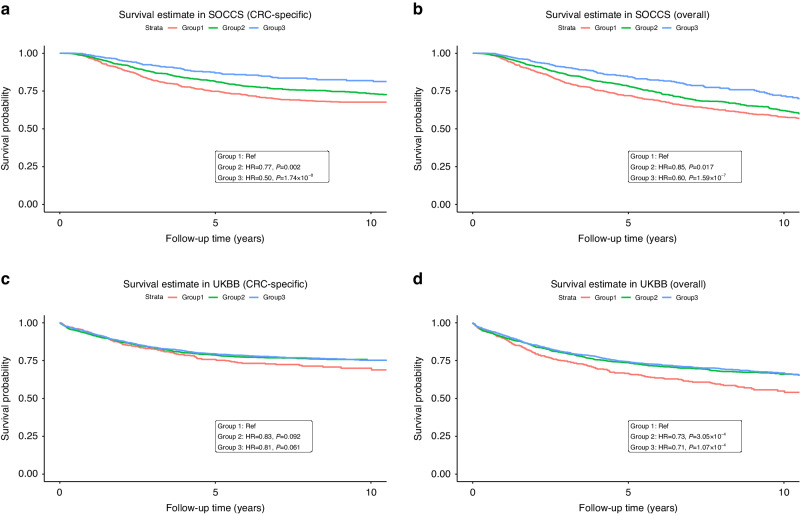
Kaplan–Meier survival estimates of colorectal cancer-specific and overall survival in different circulating 25-OHD groups. Survival estimates of colorectal cancer-specific survival **a** in SOCCS and **c** in UKBB, and overall survival **b** in SOCCS and **d** in UKBB, within different circulating 25-OHD groups. (Group 1: 25-OHD < 25 nmol/L, Group 2: 25-OHD = 25–50 nmol/L, Group 3: 25-OHD > 50 nmol/L). The HR and the corresponding *P* value were estimated in Model 1 by comparing the colorectal cancer-specific survival or overall survival difference between patients in Group 2/3 and in Group 1.

**Fig. 2:**
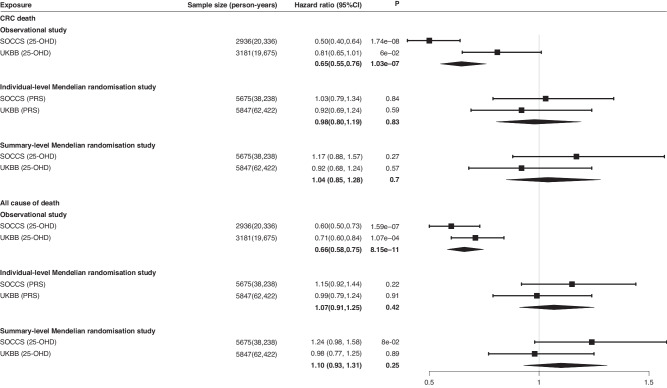
Forest plots for hazard ratios of colorectal cancer-specific and overall survival. The results of observational studies were tested in Model 1 by comparing the CCS or OS difference between patients with Group 3 and Group 1 25-OHD levels. The results of individual-level Mendelian randomisation studies were the CCS or OS difference per unit increase in 25-OHD PRS after adjusting age and sex. The results of summary-level Mendelian randomisation studies were the CCS or OS difference per standard deviation increase of rank-based inverse-normal transformed 25-OHD by adjusting age, sex and stages in SOCCS, and age and sex in UKBB.

In general, we found no evidence of significant deviation from the proportional hazard assumption for 25-OHD and observed broadly consistent time-dependent effects for the covariates (Tables S5 and S6). The Kaplan-Meier survival estimates of cancer-specific (CSS) and overall survival (OS) under three 25-OHD groups are presented in Fig. 1. Cox models were employed to estimate the effect sizes of 25-OHD on CRC survival outcomes. As shown in Table 2 and Fig. 2, we observed significantly poorer CSS and OS for patients with the lowest 25-OHD in both SOCSS and UKBB, and meta-analysis yielded an HR of 0.65 (95%CI = 0.55–0.76, *P* = 1.03 × 10^−7^) for CSS and 0.66 (95%CI = 0.58–0.75, *P* = 8.15 × 10^−11^) for OS. Consistent improvement in CSS and OS was identified in SOCCS when comparing patients in Group 2 with those in Group 1, although significant benefit was only found for OS in UKBB (Table 2).

**Table 2:** Results of Cox regression models for the effect of circulating 25-OHD on colorectal cancer survival.

	Group 1^a^	Group 2^a^			Group 3^a^			
		HR	95%CI	*P*	HR	95%CI	*P*	*P* trend
CRC death-SOCCS								
Model 1	Ref	0.77	0.65–0.90	0.002	0.50	0.40–0.64	1.74 × 10^−8^	9.87 × 10^−11^
Model 2	Ref	0.75	0.64–0.89	0.001	0.50	0.39–0.63	1.12 × 10^−8^	5.00 × 10^−11^
Model 3	Ref	0.81	0.66–0.99	0.044	0.45	0.32–0.65	1.51 × 10^−5^	1.56 × 10^−10^
All cause of death-SOCCS								
Model 1	Ref	0.85	0.74–0.97	0.017	0.60	0.50–0.73	1.59 × 10^−7^	2.99 × 10^−10^
Model 2	Ref	0.84	0.73–0.96	0.012	0.59	0.49–0.72	1.23 × 10^−7^	1.84 × 10^−10^
Model 3	Ref	0.90	0.76–1.06	0.195	0.60	0.46–0.78	1.30 × 10^−4^	1.41 × 10^−9^
CRC death- UKBB								
Model 1	Ref	0.83	0.67–1.03	0.092	0.81	0.65–1.01	0.061	0.042
Model 2	Ref	0.84	0.68–1.03	0.100	0.82	0.66–1.02	0.069	0.050
Model 3	Ref	0.85	0.69–1.06	0.149	0.83	0.67–1.04	0.110	0.070
All cause of death-UKBB								
Model 1	Ref	0.73	0.62–0.87	3.05 × 10^−4^	0.71	0.60–0.84	1.07 × 10^−4^	0.001
Model 2	Ref	0.74	0.62–0.87	4.43 × 10^−4^	0.71	0.60–0.85	1.42 × 10^−4^	0.002
Model 3	Ref	0.74	0.63–0.88	0.001	0.72	0.60–0.86	3.12 × 10^−4^	0.003

Model1: Adjusted for age and sex; Model 2: Adjusted for age, sex, tumour site (colon, rectum and colorectum), and season of blood sampling; Model 3 in SOCCS: age, sex, tumour site (colon, rectum and colorectum), the season of blood sampling and AJCC stages; Model 3 in UKBB: age, sex, tumour site (colon, rectum and colorectum), the season of blood sampling and BMI; P trend was tested in Model 1 by using the continuous rank-based inverse-normal transformed 25-OHD.

*SOCCS* Study of Colorectal Cancer in Scotland, *UKBB* UK Biobank, *HR* hazard ratio, *CI* confidence interval.

^a^Group 1: 25-OHD < 25 nmol/L, Group 2: 25OHD = 25-50 nmol/L, Group 3: 25OHD > 50 nmol/L.

The basic characteristics of CRC patients in subgroups are presented in Table S7. For colon cancer, the lower 25-OHD level was associated with poorer CSS (*P* = 6.82 × 10^−8^) and OS in SOCCS (*P* = 2.87 × 10^−7^) but not in UKBB (Table S8). For rectal cancer, the lower 25-OHD level was associated with poorer CSS in SOCCS (*P* = 2.00 × 10^−4^) and OS in both SOCCS (*P* = 2.00 × 10^−4^) and UKBB (*P* = 0.002) (Table S8). The lower 25-OHD levels were strongly associated with poorer CSS (stage II: *P* = 0.002; stage III: *P* = 4.79 × 10^−5^) and OS (stage II: *P* = 5.75 × 10^−7^; stage III: *P* = 1.78 × 10^−5^) among stage II and stage III patients in SOCCS (Table S8). Additionally, we observed a significant effect difference between GG and AA/AG genotype carriers of rs11568820 in SOCCS but not in UKBB (Table S9). In the interaction analysis of SOCCS, the interaction term had an HR of 0.82 (95%CI = 0.69–0.96, *P* = 0.014) for CCS and 0.84 (95%CI = 0.73–0.95, *P* = 0.008) for OS, taking AA/AG genotypes as the reference.

### Mendelian randomisation study

A total of 5675 and 5847 CRC patients who have genotype data with 38,238 and 62,422 person-years of follow-up in SOCCS and UKBB respectively were included in the MR analyses (Table S10). The PRS was significantly correlated with an increased vitamin D level among both CRC cases (*P* = 1.60 × 10^−8^ in SOCCS and *P* < 2 × 10^−16^ in UKBB) and non-CRC participants (*P* = 2.61 × 10^−6^ in SOCCS and *P* < 2 × 10^−16^ in UKBB; Table S11). By applying these 113 or 107 SNPs, we generated strong genetic instruments with F-statistics exceeding 343 for SOCCS and 353 for UKBB respectively (Table S12). In the meta-analysis of these two studies, our statistical power is 80% to detect a minimum HR of 0.81 for CSS and 0.84 for OS.

**Fig. 3:**
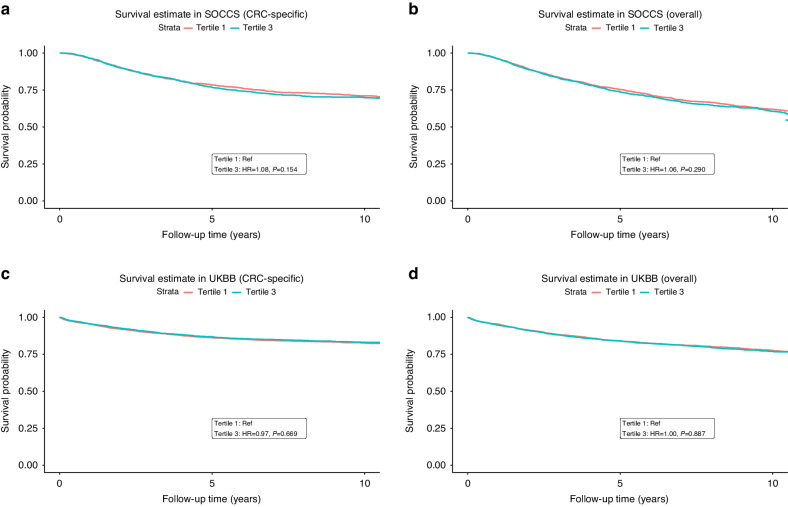
Kaplan–Meier survival estimates of colorectal cancer-specific and overall survival in the top and bottom tertiles of 25-OHD PRS. Survival estimates of colorectal cancer-specific survival **a** in SOCCS and **c** in UKBB, and overall survival **b** in SOCCS and **d** in UKBB, in the top (Tertile 3) and bottom (Tertile 1) tertiles of 25-OHD PRS. The HR and the corresponding *P* value were estimated by comparing the colorectal cancer-specific survival or overall survival difference between patients in tertile 3 and in tertile 1 of 25-OHD PRS, adjusting for age and sex.

The Kaplan-Meier survival estimates of CSS and OS by comparing tertiles of PRS are presented in Fig. 3. No significant causal association was observed between 25-OHD and CRC survival among SOCCS, UKBB or combined (Fig. 2 and Table S13). By using PRS, the HRs (95%CI) were 0.98 (0.80, 1.19) and 1.07 (0.91, 1.25) for CSS and OS respectively per unit increase in 25-OHD PRS after performing a meta-analysis of SOCCS and UKBB estimations. In the summary-level MR, similar effect estimates, namely null causal associations, were observed in the IVW model. The HRs (95%CI) were 1.04 (0.85, 1.28) and 1.10 (0.93, 1.31) for CSS and OS respectively per standard deviation increase of the rank-based inverse-normal transformed 25-OHD (Table S14, Fig. S4). The sensitivity analyses reported similar estimations. The MR-Egger test did not indicate significant horizontal pleiotropy and the Q statistic did not indicate significant heterogeneity (Table S14). Additionally, neither the VDR polymorphism (rs11568820) nor the PRS after categorising by genotypes of rs11568820 was associated with CRC survival (Table S15). Sensitivity analysis using the initial six variants identified from the SUNLIGHT GWAS did not find any causal associations between 25-OHD PRS_6_ and CRC survival either (Table S16).

## Discussion

Based on the two prospective cohorts, we observed robust associations between lower circulating 25-OHD and poorer CRC-CCS and -OS. The association was consistent across patients with tumours of various stages and anatomical sites. However, although we created a strengthened genetic instrument to perform both individual- and summary-level MR analyses, the results did not support a causal association between 25-OHD and CRC survival outcomes. We categorised patients using clinical cutouts of 25-OHD and findings were consistent with our previous publications [4, 5]. Compared to the previous studies, the sample size in SOCCS was improved and the findings were further validated in UKBB. In addition, our results were consistent with other prospective studies which suggested CRC patients with vitamin D deficiency and insufficiency are at greater risk [6, 33]. In a study from the U.S. with 304 CRC patients, the HR (95%CI) by comparing survival of patients with circulating 25-OHD level of 47–59 nmol/L and <47 nmol/L was 0.76 (0.41, 1.42) for CSS and 0.81 (0.49, 1.35) for OS [34]. Another study with 2832 CRC patients from Germany compared the survival of patients with the highest and the lowest level (>45.20 nmol/L vs <11.83 nmol/L) of circulating 25-OHD and reported an HR (95%CI) of 0.56 (0.44,0.71) for CSS and 0.60 (0.45, 0.80) for OS [6]. The effect estimates from the current study were consistent with them and statistically significant, which indicated the substantially increased statistical power of our study.

Our results indicated the effect of 25-OHD on CRC survival outcomes may be stronger in patients with later stages and those who carry the GG genotype of *Cdx-2 VDR* polymorphism. Evidence has supported a stronger effect in more advanced stages [3, 34]. In the current study, 25-OHD effects were observed strongly in stage II and stage III, and marginally in stage IV, but not in stage I patients. In addition, stage I patients had the highest median 25-OHD level while stage IV patients had the lowest. It is possible that participants with higher 25-OHD tend to undergo CRC screening more frequently which results in the earlier detection of CRC. VDR is an important factor in the regulation of calcium absorption functioned by the active form of vitamin D (1,25-dihydroxyvitamin D3). In the current study, we have replicated the interacting effect of VDR polymorphism in the enlarged SOCCS cohort while failing to replicate them in UKBB. This could be due to the difference between studies, which merits further investigation into diverse datasets.

25-OHD is the precursor to the steroid hormone calcitriol which is related to numerous mechanisms of oncogenesis [35]. For example, calcitriol is related to the induction of colonic epithelial cell differentiation in colon cancer patients through mechanisms such as the regulation of the β-catenin and transforming growth factor-β [36–38]. Calcitriol exhibits anti-inflammatory effects on tumour cells through pathways such as suppression of prostaglandin action [39] and effects related to tumour growth, invasion and angiogenesis through inhibiting the expression of tenascin-C [40]. However, we did not find a causal association between circulating 25-OHD and CRC survival by conducting MR analyses, even though, the strong genetic instrument has significantly increased the statistical power of this study.

The results of published RCTs were inconclusive. The SUNSHINE trial with 139 CRC patients receiving mFolfox6 and bevacizumab therapy plus high versus standard-dose vitamin D supplement (8000 IU/d vs 400 IU/d), found a non-significant improvement in median progression-free survival for patients with high dosage vitamin D supplement [9]. Another trial (AMATERASU) from Japan did not identify significantly improved relapse-free survival for resected epithelial carcinomas in the digestive tract (200 CRC patients) with supplementation of vitamin D (2000 IU/d) [41]. Our MR analysis added evidence that vitamin D supplementation may lead to limited benefits in terms of survival outcomes for CRC patients. It could also be possible that the lack of evidence from RCTs was due to insufficient study sample size, study duration, and dose of supplementation [42, 43]; the lack of evidence from MR studies could be due to the variance of vitamin D leveraged by genetic instruments was generally low [44]. Our previous MR analyses targeting CRC risk have challenged the protective effect of 25-OHD against carcinogenesis, which implied the effect on cancer progression could also be small [2, 45]. Given the biological plausibility and limited sample size of our MR study, further efforts are warranted to explore potential small to modest causal effects, and also effects in more targeted sub-populations of CRC patients.

Circulating 25-OHD is determined by the joint effect from genetics and environmental factors such as vitamin D supplements intake [46] which could potentially confound the observed association between 25-OHD and survival. However, our analyses were performed based on the measured concentrations of 25-OHD, and we were unable to further dissect this effect due to unavailable data. Consequently, caution is warranted in the interpretation of the results and future research is needed to investigate the role of vitamin D supplementation in this association.

### Strengths and limitations

One of the major strengths of the current study is the large sample size of the two prospective studies which increases the statistical power of this study and allows comprehensive subgroup analyses. Another strength of this study is that we tested both observational and casual associations. To test the potential causality, we applied a powerful genetic instrument. Additionally, we explored the potential genotype-specific effect of VDR polymorphism in a larger sample set. This study has limitations. First, since our analysis was subject to data unavailability, we were not able to test the associations by tumour stage in UKBB or adjust for some possible confounders, such as patient’s performance status at diagnosis [47], treatment and comorbidities (e.g., diarrhoea and neutropenia) [48] that were not measured in our cohorts. Nevertheless, these potential confounding effects could to some extent be overcome by the naturally randomised genetic instruments in the MR analyses. Second, although the newly detected genetic variants for vitamin D have remarkably increased the power of the MR analyses, limited power could be a potential limitation of MR studies. Third, even though we have applied multiple MR methods with diverse assumptions, it is not possible to entirely alleviate the concerns around the validity of the genetic instrument at present, in particular relating to the associations between genetic instruments and confounders. Finally, two cohorts of the current study were based in the UK, which could limit the generalisability of our results.

## Conclusion

In summary, in this large observational and MR study, we found a lower level of circulating 25-OHD is associated with worse CRC-specific and overall survival. The association is retained across different tumour stages and sites. The MR analyses did not support causal associations between circulating 25-OHD and CRC survival. Our findings suggest that vitamin D may serve as a prognostic biomarker, but it is less likely a possible therapeutic target for CRC patients.

### Supplementary information

Supplementary methods, tables and figures

## Data Availability

The individual-level datasets used and/or analysed during the current study are available from the corresponding author on reasonable request. All summary statistics used in this study can be found in supplementary materials.
