# Supplementary material for: Circulating 25-hydroxyvitamin D and survival outcomes of colorectal cancer: evidence from population-based prospective cohorts and Mendelian randomisation
Source: Br J Cancer. 2024 Mar 13;130(9):1585–91. doi: 10.1038/s41416-024-02643-5 (PMC11058806; doi:10.1038/s41416-024-02643-5)
Supplement: Supplementary file 1 — Supplementary methods, tables and figures [file 41416_2024_2643_MOESM1_ESM.docx]

**Title:** **Circulating 25-hydroxyvitamin D and survival outcomes of colorectal cancer: evidence from population-based prospective cohorts and Mendelian randomisation**

Authors: Xiaomeng Zhang^1^*, Yazhou He^1,3^*, Xue Li^1,4^, Rasha Shraim^5^, Wei Xu^1^, Lijuan Wang^1^, Susan M Farrington^2^, Harry Campbell^1^, Maria Timofeeva^6^, Lina Zgaga^5^, Peter Vaughan-Shaw^2^, Evropi Theodoratou^1,2#*^, Malcolm G Dunlop^2#*^

1. Centre for Global Health, Usher Institute, University of Edinburgh, UK
2. Cancer Research UK Edinburgh Centre, Medical Research Council Institute of Genetics & Molecular Medicine, Western General Hospital, The University of Edinburgh, Edinburgh, UK
3. Department of Oncology, West China School of Public Health and West China Fourth Hospital, Sichuan University, Chengdu, China
4. School of Public Health and the Second Affiliated Hospital, Zhejiang University, Hangzhou, China
5. Department of Public Health and Primary Care, Institute of Population Health, Trinity College Dublin, Dublin, Republic of Ireland
6. Danish Institute for Advanced Study (DIAS), Department of Public Health, University of Southern Denmark, Odense, Denmark

*These authors contributed equally to this work. The authors declare no potential conflicts of interest.

#Correspondence author

Correspondence:

Prof. Malcolm G. Dunlop, Institute of Genetics and Molecular Medicine, The University of Edinburgh, Western General Hospital, Edinburgh EH4 2XU, UK, Tel +44 (0) 131 651 8631, Email address: [malcolm.dunlop@ed.ac.uk](mailto:malcolm.dunlop@ed.ac.uk)

Prof. Evropi Theodoratou, Centre for Global Health Research, Usher Institute, University of Edinburgh, Teviot Place, Edinburgh, EH8 9AG, United Kingdom; Tel: (+44) 131-650-6194; E-mail: [E.Theodoratou@ed.ac.uk](mailto:E.Theodoratou@ed.ac.uk)

Contents

[Section I: Supplementary methods 3](#_Toc159425292)

[Datasets 3](#_Toc159425293)

[Genetic proxies for vitamin D 4](#_Toc159425294)

[Section II: Supplementary tables and figures 4](#_Toc159425295)

[Table S1 Instrumental variable for vitamin D in SOCCS 4](#_Toc159425296)

[Table S2 Instrumental variable for vitamin D in UKBB 7](#_Toc159425297)

[Table S3 Summarised characteristics of colorectal cancer patients by 25-OHD groups in SOCCS (n=2936) 9](#_Toc159425298)

[Table S4 Summarised characteristics of colorectal cancer patients by 25-OHD groups in UKBB (n=3181) 9](#_Toc159425299)

[Table S5 Schoenfeld residuals for the Cox regression models before and after categorising data into subgroups in SOCCS 10](#_Toc159425300)

[Table S6 Schoenfeld residuals for the Cox regression model before and after categorising data into subgroups in UKBB 11](#_Toc159425301)

[Table S7 Summarised characteristics of colorectal cancer patients by subgroups 12](#_Toc159425302)

[Table S8 Observational analyses for the association between 25-OHD and colorectal cancer survival in SOCCS and UKBB by stages and tumour sites 12](#_Toc159425303)

[Table S9 Observational analyses for the association between 25-OHD and colorectal cancer survival in SOCCS and UKBB by rs11568820 genotypes 13](#_Toc159425304)

[Table S10 Summarised characteristics of colorectal cancer patients in the Mendelian randomisation study 14](#_Toc159425305)

[Table S11 Performance of 25-OHD polygenic risk score in SOCCS and UK Biobank 14](#_Toc159425306)

[Table S12 Power estimation 15](#_Toc159425307)

[Table S13 Results of individual-level MR analyses for effect of rank-based inverse-normal transformed circulating 25-OHD on colorectal cancer survival in SOCCS and UKBB 15](#_Toc159425308)

[Table S14 Results of summary-level MR analyses for effect of rank-based inverse-normal transformed circulating 25-OHD on colorectal cancer survival in SOCCS and UKBB 16](#_Toc159425309)

[Table S15 Results of individual-level MR analyses by taking rs11568820 or PRS after categorising based on rs11568820 as genetic instrument to colorectal cancer survival in SOCCS and UKBB 17](#_Toc159425310)

[Table S16 MR results between vitamin D and CRC survival using six vitamin D related loci detected by SUNLIGHT GWAS 17](#_Toc159425311)

[Figure S1 Flow chart of Vitamin D genetic variants selection 18](#_Toc159425312)

[Figure S2 Predicted hazard ratio by May-standardised 25-OHD in SOCCS and UKBB 18](#_Toc159425313)

[Figure S3 Kaplan-Meier survival estimates of colorectal cancer-specific (A: SOCCS; C: UKBB) and overall survival (B: SOCCS; D: UKBB) among incident and prevalent cases 19](#_Toc159425314)

[Figure S4 Scatter plots for summary-level Mendelian randomisation analyses 19](#_Toc159425315)

# Section I: Supplementary methods

## Datasets

The Study of Colorectal Cancer in Scotland (SOCCS) is a prospective, population-based case-control study aiming to investigate genetic and environmental factors associated with risk and survival of newly diagnosed CRC cases across Scotland.^1^ This study received approvals from the MultiCentre Research Ethics Committee for Scotland, local research ethics committees, and National Health Service management. All participants provided informed written consent. Survival outcomes of all participants were obtained through linkage to the Scottish national death records. The primary cause of death (CRC and all-cause) was collected independently by two researchers (L.Z., F.V.N.D) with a 99% concordance. Survival time was calculated from the time of CRC diagnosis to death or the date of last follow-up (July 1st, 2017). Age and sex were collected at the recruitment through questionnaire. Tumour site, multiplicity and clinicopathologic staging were obtained from the clinical or tumour boards. Preoperative imaging was also collected. Tumour stage was assigned to each patient by estimating the collected pathology, imaging, and clinical information according to the Union for International Cancer Control TNM staging system and was mapped onto the American Joint Committee on Cancer (AJCC) staging system (AJCC stage I to IV). The plasma 25-OHD (25-OHD2 and 25-OHD3) level of participants was measured by liquid chromatography-tandem mass spectrometry.^2^ To minimize the influence of technical variability, the plasma samples were batched and the 25-OHD was assayed based on a predefined protocol in the same laboratory.

The UK Biobank is a large-scale prospective cohort with in-depth genetic and health information for volunteers aged 40 to 69 years old from the general population of the UK.^3^ The UKBB cohort received approvals from the North West Multi-centre Research Ethics Committee (11/NW/0382). Informed consent was signed by the participants at recruitment. Cancer diagnosis and survival outcomes of CRC patients were obtained from the Cancer Registry data and Death Registry data respectively. Demographic information was collected at recruitment through questionnaires. Tumour sites were also extracted from the Cancer Registry data. The follow-up time was calculated as years from the date of cancer diagnosis to death or the censoring date (January 24^th^, 2021). The total 25-OHD concentration (i.e., 25-OHD2 and 25-OHD3) was measured by using the DiaSorin Liaison XL assay in samples collected at baseline (2006-2010) and a repeat assessment (2012-2013). A total of 449,913 participants in the UKBB has 25-OHD concentration records. In the present study, we used baseline measures of 25-OHD from 448,311 participants. For the genotype data, genotyping, quality control and genotype imputation were conducted by the UK Biobank team before the data release and the procedure is described by Bycroft et al^3^. The initial 50 000 participants were genotyped by the Affymetrix UK BiLEVE Axiom array and the remaining 450 000 participants were genotyped by the Affymetrix UK Biobank Axiom array. Genotype imputation was performed using a merged reference panel of the Haplotype Reference Consortium (HRC)^4^ and the UK10K haplotype resources,^5^ and the classical allelic variations at the MHC region were further imputed by using an additional multi-population reference panel^6^. For study specific quality control, a list of field variables was made available by the UK Biobank to indicate the genotype quality, population structure, and genetic relatedness.

The circulating 25-OHD level was May-standardised by adjusting for sampling month using the difference in age- and sex-standardised monthly average obtained from non-CRC cases in SOCCS and UKBB respectively ^7, 8^. The rank-based inverse-normal transformation was applied to normalise the distribution of May-standardised 25-OHD levels.

## Genetic proxies for vitamin D

A total of 133 genetic variants associated with circulating 25-OHD concentration at p<5x10^-8^ were selected based on findings from the latest genome-wide association study (GWAS) with 417,580 Europeans from the UKBB ^9^. The variance of 25-OHD level explained by these genetic variants was about 5.7% to 10.5% ^9^. To generate the genetic instrument for 25-OHD, we first checked the availability of these variants in SOCCS and UKBB. For variants that were not present in either of our study cohorts, a proxy variant in strong linkage disequilibrium (LD, R^2^>0.8) with the missing variant was identified based on the 1000 Genomes reference panel. We excluded variants following the criteria described in **Figure S1**. At last, 113 variants in UKBB and 107 variants in SOCCS were retained for subsequent analyses. To perform individual-level MR, we generated a polygenic risk score (PRS) as a proxy for 25-OHD by adding the weighted (by the effect estimate of each variant) dosages of risk alleles for each of the variants. The list of genetic variants for 25-OHD is presented in **Table S1& Table S2**.

# Section II: Supplementary tables and figures

## Table S1 Instrumental variable for vitamin D in SOCCS

| **rsid** | **Chr** | **Position** | **Effect allele** | **Other allele** | **EAF** | **Beta** | **SE** | **P** |
| --- | --- | --- | --- | --- | --- | --- | --- | --- |
| rs10070734 | 5 | 87940026 | T | C | 0.290 | -0.013 | 0.002 | 1.82E-09 |
| rs10083762* | 16 | 11907210 | C | G | 0.727 | -0.014 | 0.002 | 1.48E-09 |
| rs10085881 | 7 | 21577960 | T | C | 0.718 | 0.015 | 0.002 | 7.83E-11 |
| rs1038165 | 12 | 68665940 | C | T | 0.417 | -0.012 | 0.002 | 2.31E-09 |
| rs10426 | 19 | 51517798 | G | A | 0.787 | -0.026 | 0.002 | 4.64E-26 |
| rs10454087 | 17 | 40735641 | C | T | 0.715 | 0.014 | 0.002 | 8.70E-10 |
| rs1047891 | 2 | 211540507 | C | A | 0.684 | 0.015 | 0.002 | 1.18E-12 |
| rs10859995# | 12 | 96375682 | T | C | 0.417 | 0.040 | 0.002 | 1.05E-88 |
| rs10887718 | 10 | 82042624 | C | T | 0.472 | 0.011 | 0.002 | 2.61E-08 |
| rs10908419 | 1 | 154567699 | G | A | 0.51 | 0.012 | 0.002 | 5.81E-10 |
| rs10908465 | 1 | 155389688 | C | T | 0.73 | -0.017 | 0.002 | 6.12E-14 |
| rs11076175 | 16 | 57006378 | A | G | 0.822 | -0.023 | 0.003 | 9.47E-19 |
| rs11182428 | 12 | 38526387 | T | C | 0.480 | 0.013 | 0.002 | 3.23E-10 |
| rs11295714* | 14 | 104245948 | CA | C | 0.653 | 0.013 | 0.002 | 6.69E-10 |
| rs1149605 | 11 | 76485216 | T | C | 0.830 | -0.022 | 0.003 | 1.31E-16 |
| rs11606 | 19 | 54658102 | C | G | 0.575 | -0.012 | 0.002 | 4.41E-09 |
| rs11721204* | 3 | 49982765 | C | T | 0.562 | -0.014 | 0.002 | 1.17E-11 |
| rs11732896 | 4 | 88287993 | G | A | 0.701 | 0.016 | 0.002 | 1.79E-13 |
| rs12056768 | 8 | 116988527 | T | G | 0.417 | 0.023 | 0.002 | 6.44E-31 |
| rs12317268 | 12 | 21352541 | A | G | 0.849 | 0.021 | 0.003 | 6.19E-14 |
| rs12372115 | 12 | 97982701 | G | T | 0.929 | 0.022 | 0.004 | 1.93E-08 |
| rs1260326 | 2 | 27730940 | T | C | 0.393 | -0.021 | 0.002 | 4.41E-24 |
| rs12794714# | 11 | 14913575 | G | A | 0.578 | 0.088 | 0.002 | 0.00E+00 |
| rs12881545 | 14 | 101176212 | G | C | 0.327 | -0.012 | 0.002 | 2.91E-08 |
| rs13284054 | 9 | 107669073 | T | C | 0.882 | -0.018 | 0.003 | 2.07E-08 |
| rs1352846# | 4 | 72617775 | A | G | 0.709 | 0.193 | 0.002 | 0.00E+00 |
| rs142158911 | 19 | 11190534 | G | A | 0.885 | -0.026 | 0.003 | 4.79E-16 |
| rs1660839# | 11 | 71094232 | G | A | 0.751 | -0.029 | 0.002 | 6.40E-37 |
| rs17216707# | 20 | 52732362 | T | C | 0.817 | 0.038 | 0.003 | 3.47E-46 |
| rs17231506 | 16 | 56994528 | C | T | 0.677 | 0.018 | 0.002 | 5.45E-18 |
| rs1800588 | 15 | 58723675 | C | T | 0.785 | 0.033 | 0.002 | 4.38E-42 |
| rs1933064 | 1 | 152301576 | G | A | 0.53 | -0.016 | 0.002 | 9.80E-15 |
| rs2012736 | 2 | 234622379 | C | A | 0.919 | 0.048 | 0.004 | 1.16E-39 |
| rs2037511 | 18 | 61366207 | G | A | 0.834 | -0.018 | 0.003 | 1.35E-11 |
| rs2074735 | 22 | 31535872 | G | C | 0.936 | -0.028 | 0.004 | 8.23E-12 |
| rs212100 | 19 | 48376995 | T | C | 0.164 | 0.066 | 0.003 | 1.61E-133 |
| rs2131925 | 1 | 63025942 | G | T | 0.36 | 0.023 | 0.002 | 3.61E-28 |
| rs2186777* | 11 | 71219803 | C | A | 0.169 | -0.110 | 0.003 | 0.00E+00 |
| rs2229742 | 21 | 16339172 | G | C | 0.897 | 0.025 | 0.003 | 1.48E-14 |
| rs2248551 | 6 | 131924689 | G | A | 0.835 | 0.023 | 0.003 | 3.04E-18 |
| rs2346264 | 7 | 133536351 | A | C | 0.217 | 0.014 | 0.002 | 1.20E-08 |
| rs2365528* | 4 | 72758536 | A | G | 0.275 | 0.054 | 0.002 | 7.73E-129 |
| rs2520276* | 7 | 107129404 | C | T | 0.510 | 0.011 | 0.002 | 3.58E-08 |
| rs2585442 | 20 | 52737123 | C | G | 0.759 | -0.036 | 0.002 | 6.70E-51 |
| rs261291 | 15 | 58680178 | T | C | 0.645 | 0.027 | 0.002 | 2.50E-39 |
| rs2710651 | 2 | 63166379 | G | A | 0.472 | 0.011 | 0.002 | 9.51E-09 |
| rs2847500 | 11 | 120114421 | G | A | 0.876 | 0.022 | 0.003 | 4.42E-13 |
| rs28692966 | 8 | 25892919 | G | A | 0.747 | -0.015 | 0.002 | 1.13E-10 |
| rs2952289 | 17 | 66464414 | C | T | 0.202 | -0.018 | 0.002 | 1.18E-12 |
| rs31612 | 5 | 108996643 | T | C | 0.826 | 0.015 | 0.003 | 4.15E-08 |
| rs325384 | 15 | 100229761 | C | T | 0.716 | 0.014 | 0.002 | 1.66E-10 |
| rs35408430 | 1 | 17560195 | C | T | 0.658 | 0.021 | 0.002 | 1.36E-24 |
| rs3849374 | 2 | 101443397 | G | C | 0.822 | 0.016 | 0.003 | 7.46E-10 |
| rs3925446 | 10 | 91495322 | G | A | 0.801 | -0.015 | 0.002 | 1.09E-09 |
| rs4121823 | 18 | 47144223 | T | A | 0.155 | 0.019 | 0.003 | 3.83E-12 |
| rs4327060 | 16 | 72807438 | C | T | 0.946 | 0.024 | 0.004 | 2.92E-08 |
| rs4364259 | 4 | 15892159 | G | A | 0.798 | -0.016 | 0.003 | 2.16E-10 |
| rs4418728 | 10 | 94839724 | G | T | 0.548 | -0.011 | 0.002 | 4.24E-08 |
| rs4495172* | 5 | 148114622 | C | A | 0.565 | -0.011 | 0.002 | 1.94E-07 |
| rs4575545 | 16 | 79755446 | G | A | 0.695 | 0.016 | 0.002 | 7.47E-13 |
| rs4616820 | 4 | 57745481 | C | T | 0.535 | 0.012 | 0.002 | 1.13E-09 |
| rs4738684 | 8 | 59393273 | A | G | 0.334 | -0.012 | 0.002 | 4.41E-09 |
| rs541041 | 2 | 21294975 | G | A | 0.181 | 0.015 | 0.003 | 2.27E-09 |
| rs55829990 | 15 | 63790642 | T | C | 0.656 | 0.019 | 0.002 | 9.12E-19 |
| rs58542926* | 19 | 19379549 | C | T | 0.924 | -0.040 | 0.004 | 6.87E-26 |
| rs590215 | 18 | 57904088 | C | T | 0.734 | 0.013 | 0.002 | 1.04E-08 |
| rs6003456 | 22 | 23356100 | T | A | 0.765 | 0.013 | 0.002 | 1.94E-08 |
| rs6123359 | 20 | 52714706 | A | G | 0.898 | -0.034 | 0.003 | 6.10E-25 |
| rs61891388 | 11 | 66079818 | T | G | 0.544 | -0.013 | 0.002 | 4.06E-10 |
| rs62007299 | 15 | 77711719 | G | A | 0.287 | 0.013 | 0.002 | 1.32E-09 |
| rs6438900* | 3 | 125148287 | C | G | 0.742 | -0.014 | 0.002 | 3.07E-09 |
| rs6520168* | 22 | 50856938 | A | C | 0.673 | 0.012 | 0.002 | 1.26E-08 |
| rs666720* | 1 | 46020795 | T | C | 0.568 | -0.010 | 0.002 | 5.13E-07 |
| rs6671730 | 1 | 2339139 | G | A | 0.566 | 0.015 | 0.002 | 1.92E-13 |
| rs6672758 | 1 | 230303512 | C | T | 0.199 | -0.018 | 0.003 | 2.40E-12 |
| rs6782190 | 3 | 85639672 | G | A | 0.352 | 0.017 | 0.002 | 1.45E-16 |
| rs6800637* | 3 | 18775592 | T | A | 0.281 | 0.012 | 0.002 | 2.62E-08 |
| rs6966728 | 7 | 104618318 | C | T | 0.537 | 0.012 | 0.002 | 8.01E-09 |
| rs7001588* | 8 | 61353688 | G | A | 0.870 | 0.017 | 0.003 | 7.05E-09 |
| rs705117 | 4 | 72608115 | C | T | 0.148 | 0.033 | 0.003 | 1.06E-32 |
| rs71166630* | 19 | 11918093 | AT | A | 0.629 | 0.012 | 0.002 | 2.55E-09 |
| rs7149014 | 14 | 29802911 | T | C | 0.371 | 0.013 | 0.002 | 5.42E-10 |
| rs727857 | 2 | 58981967 | G | A | 0.389 | 0.014 | 0.002 | 1.05E-11 |
| rs72834856 | 6 | 22801858 | T | G | 0.928 | 0.025 | 0.004 | 8.67E-11 |
| rs72997623 | 11 | 75488054 | C | A | 0.915 | -0.028 | 0.004 | 1.25E-14 |
| rs73413596 | 12 | 111582630 | T | C | 0.926 | -0.022 | 0.004 | 1.41E-08 |
| rs7412 | 19 | 45412079 | C | T | 0.918 | -0.030 | 0.004 | 1.36E-16 |
| rs7522116 | 1 | 41835685 | C | T | 0.434 | 0.013 | 0.002 | 2.97E-11 |
| rs7528419 | 1 | 109817192 | A | G | 0.775 | -0.020 | 0.002 | 1.35E-16 |
| rs7569755 | 2 | 118648261 | G | A | 0.709 | -0.014 | 0.002 | 1.18E-10 |
| rs75741381 | 7 | 100809458 | C | G | 0.852 | 0.017 | 0.003 | 4.15E-09 |
| rs7699955* | 4 | 69947737 | G | C | 0.522 | 0.032 | 0.002 | 6.33E-57 |
| rs77532868 | 10 | 88081438 | C | T | 0.946 | -0.027 | 0.004 | 1.57E-09 |
| rs7784802 | 7 | 64015379 | A | T | 0.639 | -0.014 | 0.002 | 2.62E-11 |
| rs77924615 | 16 | 20392332 | G | A | 0.807 | 0.017 | 0.003 | 7.11E-11 |
| rs78151190 | 6 | 25619007 | A | C | 0.871 | 0.017 | 0.003 | 1.39E-08 |
| rs78649910 | 4 | 3482213 | T | A | 0.894 | 0.021 | 0.003 | 7.15E-11 |
| rs8018720# | 14 | 39556185 | G | C | 0.177 | 0.038 | 0.003 | 1.26E-47 |
| rs804281 | 8 | 11611865 | A | G | 0.416 | -0.013 | 0.002 | 4.72E-11 |
| rs8063565* | 16 | 30883965 | G | C | 0.266 | -0.015 | 0.002 | 5.57E-11 |
| rs8091117 | 18 | 28919794 | C | A | 0.935 | 0.026 | 0.004 | 5.98E-11 |
| rs8113404 | 19 | 53065579 | C | T | 0.695 | -0.012 | 0.002 | 2.07E-08 |
| rs867772 | 1 | 220972343 | A | G | 0.315 | 0.015 | 0.002 | 1.14E-11 |
| rs9476310 | 6 | 57767576 | C | T | 0.489 | -0.012 | 0.002 | 4.21E-09 |
| rs9490317 | 6 | 121859499 | T | C | 0.554 | -0.011 | 0.002 | 3.95E-08 |
| rs964184 | 11 | 116648917 | G | C | 0.132 | -0.043 | 0.003 | 1.09E-48 |
| rs9861009 | 3 | 141654685 | T | C | 0.272 | -0.014 | 0.002 | 4.86E-10 |

*: Proxy applied. EAF: effect allele frequency; SE: standard error; #: variants corresponded to loci detected by SUNLIGHT GWAS.

## Table S2 Instrumental variable for vitamin D in UKBB

| **rsid** | **Chr** | **Position** | **Effect allele** | **Other allele** | **EAF** | **Beta** | **SE** | **P** |
| --- | --- | --- | --- | --- | --- | --- | --- | --- |
| rs10070734 | 5 | 87940026 | T | C | 0.29 | -0.013 | 0.002 | 1.8E-09 |
| rs10085881 | 7 | 21577960 | T | C | 0.72 | 0.015 | 0.002 | 7.8E-11 |
| rs1038165 | 12 | 68665940 | C | T | 0.42 | -0.012 | 0.002 | 2.3E-09 |
| rs10426 | 19 | 51517798 | G | A | 0.79 | -0.026 | 0.002 | 4.6E-26 |
| rs10454087 | 17 | 40735641 | C | T | 0.72 | 0.014 | 0.002 | 8.7E-10 |
| rs1047891 | 2 | 211540507 | C | A | 0.68 | 0.015 | 0.002 | 1.2E-12 |
| rs10642047 | 3 | 18794313 | A | ATTTC | 0.28 | 0.012 | 0.002 | 2.1E-08 |
| rs10859995# | 12 | 96375682 | T | C | 0.42 | 0.040 | 0.002 | 1.1E-88 |
| rs10887718 | 10 | 82042624 | C | T | 0.47 | 0.011 | 0.002 | 2.6E-08 |
| rs10908419 | 1 | 154567699 | G | A | 0.51 | 0.012 | 0.002 | 5.8E-10 |
| rs10908465 | 1 | 155389688 | C | T | 0.73 | -0.017 | 0.002 | 6.1E-14 |
| rs11076175 | 16 | 57006378 | A | G | 0.82 | -0.023 | 0.003 | 9.5E-19 |
| rs11182428 | 12 | 38526387 | T | C | 0.48 | 0.013 | 0.002 | 3.2E-10 |
| rs112943319 | 1 | 46015535 | A | ATATCTG | 0.58 | -0.012 | 0.002 | 1.6E-08 |
| rs11458206 | 3 | 125125159 | T | TG | 0.73 | -0.014 | 0.002 | 1.0E-09 |
| rs1149605 | 11 | 76485216 | T | C | 0.83 | -0.022 | 0.003 | 1.3E-16 |
| rs11542462 | 16 | 82033810 | G | A | 0.87 | 0.023 | 0.003 | 1.3E-15 |
| rs115621755 | 22 | 50853134 | C | T | 0.67 | 0.012 | 0.002 | 4.8E-09 |
| rs11606 | 19 | 54658102 | C | G | 0.57 | -0.012 | 0.002 | 4.4E-09 |
| rs11721204* | 3 | 49982765 | C | T | 0.56 | -0.014 | 0.002 | 1.2E-11 |
| rs11732896 | 4 | 88287993 | G | A | 0.70 | 0.016 | 0.002 | 1.8E-13 |
| rs12056768 | 8 | 116988527 | T | G | 0.42 | 0.023 | 0.002 | 6.4E-31 |
| rs12317268 | 12 | 21352541 | A | G | 0.85 | 0.021 | 0.003 | 6.2E-14 |
| rs12372115 | 12 | 97982701 | G | T | 0.93 | 0.022 | 0.004 | 1.9E-08 |
| rs1260326 | 2 | 27730940 | T | C | 0.39 | -0.021 | 0.002 | 4.4E-24 |
| rs12794714# | 11 | 14913575 | G | A | 0.58 | 0.088 | 0.002 | 0.0E+00 |
| rs12798050# | 11 | 71223256 | C | T | 0.17 | -0.110 | 0.003 | 0.0E+00 |
| rs12881545 | 14 | 101176212 | G | C | 0.33 | -0.012 | 0.002 | 2.9E-08 |
| rs13284054 | 9 | 107669073 | T | C | 0.88 | -0.018 | 0.003 | 2.1E-08 |
| rs1352846# | 4 | 72617775 | A | G | 0.71 | 0.193 | 0.002 | 0.0E+00 |
| rs138385079 | 16 | 11906659 | T | TG | 0.73 | -0.014 | 0.002 | 1.2E-09 |
| rs139148694 | 4 | 72770563 | G | GTGCTTTTATCAA | 0.28 | 0.054 | 0.002 | 1.2E-128 |
| rs142158911 | 19 | 11190534 | G | A | 0.89 | -0.026 | 0.003 | 4.8E-16 |
| rs1660839 | 11 | 71094232 | G | A | 0.75 | -0.029 | 0.002 | 6.4E-37 |
| rs17216707# | 20 | 52732362 | T | C | 0.82 | 0.038 | 0.003 | 3.5E-46 |
| rs17231506 | 16 | 56994528 | C | T | 0.68 | 0.018 | 0.002 | 5.4E-18 |
| rs1800588 | 15 | 58723675 | C | T | 0.78 | 0.033 | 0.002 | 4.4E-42 |
| rs1933064 | 1 | 152301576 | G | A | 0.53 | -0.016 | 0.002 | 9.8E-15 |
| rs200210321 | 19 | 19393890 | A | AG | 0.93 | -0.041 | 0.004 | 1.7E-26 |
| rs2012736 | 2 | 234622379 | C | A | 0.92 | 0.048 | 0.004 | 1.2E-39 |
| rs2037511 | 18 | 61366207 | G | A | 0.83 | -0.018 | 0.003 | 1.4E-11 |
| rs2074735 | 22 | 31535872 | G | C | 0.94 | -0.028 | 0.004 | 8.2E-12 |
| rs212100 | 19 | 48376995 | T | C | 0.16 | 0.066 | 0.003 | 1.6E-133 |
| rs2131925 | 1 | 63025942 | G | T | 0.36 | 0.023 | 0.002 | 3.6E-28 |
| rs2229742 | 21 | 16339172 | G | C | 0.90 | 0.025 | 0.003 | 1.5E-14 |
| rs2248551 | 6 | 131924689 | G | A | 0.83 | 0.023 | 0.003 | 3.0E-18 |
| rs2346264 | 7 | 133536351 | A | C | 0.22 | 0.014 | 0.002 | 1.2E-08 |
| rs2585442 | 20 | 52737123 | C | G | 0.76 | -0.036 | 0.002 | 6.7E-51 |
| rs261291 | 15 | 58680178 | T | C | 0.64 | 0.027 | 0.002 | 2.5E-39 |
| rs2710651 | 2 | 63166379 | G | A | 0.47 | 0.011 | 0.002 | 9.5E-09 |
| rs2762943 | 20 | 52790786 | T | G | 0.08 | -0.046 | 0.004 | 2.1E-34 |
| rs28374650 | 6 | 32623367 | C | T | 0.76 | 0.014 | 0.002 | 5.4E-09 |
| rs2847500 | 11 | 120114421 | G | A | 0.88 | 0.022 | 0.003 | 4.4E-13 |
| rs28692966 | 8 | 25892919 | G | A | 0.75 | -0.015 | 0.002 | 1.1E-10 |
| rs2952289 | 17 | 66464414 | C | T | 0.20 | -0.018 | 0.002 | 1.2E-12 |
| rs31612 | 5 | 108996643 | T | C | 0.83 | 0.015 | 0.003 | 4.2E-08 |
| rs325384 | 15 | 100229761 | C | T | 0.72 | 0.014 | 0.002 | 1.7E-10 |
| rs34407256 | 4 | 69968825 | A | AT | 0.52 | 0.032 | 0.002 | 8.0E-57 |
| rs35408430 | 1 | 17560195 | C | T | 0.66 | 0.021 | 0.002 | 1.4E-24 |
| rs368637654 | 4 | 69591271 | G | GT | 0.86 | 0.022 | 0.003 | 1.1E-14 |
| rs3814995 | 19 | 36342212 | C | T | 0.69 | 0.013 | 0.002 | 5.2E-09 |
| rs3849374 | 2 | 101443397 | G | C | 0.82 | 0.016 | 0.003 | 7.5E-10 |
| rs3925446 | 10 | 91495322 | G | A | 0.80 | -0.015 | 0.002 | 1.1E-09 |
| rs4121823 | 18 | 47144223 | T | A | 0.15 | 0.019 | 0.003 | 3.8E-12 |
| rs4327060 | 16 | 72807438 | C | T | 0.95 | 0.024 | 0.004 | 2.9E-08 |
| rs4364259 | 4 | 15892159 | G | A | 0.80 | -0.016 | 0.003 | 2.2E-10 |
| rs4418728 | 10 | 94839724 | G | T | 0.55 | -0.011 | 0.002 | 4.2E-08 |
| rs4495172* | 5 | 148114622 | C | A | 0.57 | -0.011 | 0.002 | 1.9E-07 |
| rs4575545 | 16 | 79755446 | G | A | 0.70 | 0.016 | 0.002 | 7.5E-13 |
| rs4616820 | 4 | 57745481 | C | T | 0.54 | 0.012 | 0.002 | 1.1E-09 |
| rs4738684 | 8 | 59393273 | A | G | 0.33 | -0.012 | 0.002 | 4.4E-09 |
| rs484195 | 19 | 45421877 | A | G | 0.38 | 0.016 | 0.002 | 1.4E-13 |
| rs541041 | 2 | 21294975 | G | A | 0.18 | 0.015 | 0.003 | 2.3E-09 |
| rs556230166 | 7 | 107136397 | A | AT | 0.51 | -0.012 | 0.002 | 7.9E-09 |
| rs55829990 | 15 | 63790642 | T | C | 0.66 | 0.019 | 0.002 | 9.1E-19 |
| rs590215 | 18 | 57904088 | C | T | 0.73 | 0.013 | 0.002 | 1.0E-08 |
| rs6003456 | 22 | 23356100 | T | A | 0.77 | 0.013 | 0.002 | 1.9E-08 |
| rs6123359 | 20 | 52714706 | A | G | 0.90 | -0.034 | 0.003 | 6.1E-25 |
| rs61891388 | 11 | 66079818 | T | G | 0.54 | -0.013 | 0.002 | 4.1E-10 |
| rs62007299 | 15 | 77711719 | G | A | 0.29 | 0.013 | 0.002 | 1.3E-09 |
| rs6671730 | 1 | 2339139 | G | A | 0.57 | 0.015 | 0.002 | 1.9E-13 |
| rs6672758 | 1 | 230303512 | C | T | 0.20 | -0.018 | 0.003 | 2.4E-12 |
| rs6782190 | 3 | 85639672 | G | A | 0.35 | 0.017 | 0.002 | 1.5E-16 |
| rs6966728 | 7 | 104618318 | C | T | 0.54 | 0.012 | 0.002 | 8.0E-09 |
| rs705117 | 4 | 72608115 | C | T | 0.15 | 0.033 | 0.003 | 1.1E-32 |
| rs7149014 | 14 | 29802911 | T | C | 0.37 | 0.013 | 0.002 | 5.4E-10 |
| rs727857 | 2 | 58981967 | G | A | 0.39 | 0.014 | 0.002 | 1.0E-11 |
| rs72834856 | 6 | 22801858 | T | G | 0.93 | 0.025 | 0.004 | 8.7E-11 |
| rs72997623 | 11 | 75488054 | C | A | 0.92 | -0.028 | 0.004 | 1.2E-14 |
| rs73413596 | 12 | 111582630 | T | C | 0.93 | -0.022 | 0.004 | 1.4E-08 |
| rs7412 | 19 | 45412079 | C | T | 0.92 | -0.030 | 0.004 | 1.4E-16 |
| rs7522116 | 1 | 41835685 | C | T | 0.43 | 0.013 | 0.002 | 3.0E-11 |
| rs7528419 | 1 | 109817192 | A | G | 0.78 | -0.020 | 0.002 | 1.4E-16 |
| rs7569755 | 2 | 118648261 | G | A | 0.71 | -0.014 | 0.002 | 1.2E-10 |
| rs75741381 | 7 | 100809458 | C | G | 0.85 | 0.017 | 0.003 | 4.2E-09 |
| rs763058201 | 16 | 30884552 | GTTGA | G | 0.27 | -0.015 | 0.002 | 4.4E-11 |
| rs769249577 | 8 | 61388083 | CCTG | C | 0.87 | 0.018 | 0.003 | 1.5E-09 |
| rs77532868 | 10 | 88081438 | C | T | 0.95 | -0.027 | 0.004 | 1.6E-09 |
| rs7784802 | 7 | 64015379 | A | T | 0.64 | -0.014 | 0.002 | 2.6E-11 |
| rs77924615 | 16 | 20392332 | G | A | 0.81 | 0.017 | 0.003 | 7.1E-11 |
| rs78151190 | 6 | 25619007 | A | C | 0.87 | 0.017 | 0.003 | 1.4E-08 |
| rs78649910 | 4 | 3482213 | T | A | 0.89 | 0.021 | 0.003 | 7.2E-11 |
| rs8018720# | 14 | 39556185 | G | C | 0.18 | 0.038 | 0.003 | 1.3E-47 |
| rs804281 | 8 | 11611865 | A | G | 0.42 | -0.013 | 0.002 | 4.7E-11 |
| rs8091117 | 18 | 28919794 | C | A | 0.93 | 0.026 | 0.004 | 6.0E-11 |
| rs8113404 | 19 | 53065579 | C | T | 0.70 | -0.012 | 0.002 | 2.1E-08 |
| rs867772 | 1 | 220972343 | A | G | 0.32 | 0.015 | 0.002 | 1.1E-11 |
| rs9476310 | 6 | 57767576 | C | T | 0.49 | -0.012 | 0.002 | 4.2E-09 |
| rs9490317 | 6 | 121859499 | T | C | 0.55 | -0.011 | 0.002 | 4.0E-08 |
| rs964184 | 11 | 116648917 | G | C | 0.13 | -0.043 | 0.003 | 1.1E-48 |
| rs9861009 | 3 | 141654685 | T | C | 0.27 | -0.014 | 0.002 | 4.9E-10 |
|  | 14 | 104245948 | CA | C | 0.65 | 0.013 | 0.002 | 6.7E-10 |
|  | 19 | 11918093 | AT | A | 0.63 | 0.012 | 0.002 | 2.5E-09 |

*: Proxy applied. EAF: effect allele frequency; SE: standard error, #: variants corresponded to loci detected by SUNLIGHT GWAS.

## Table S3 Summarised characteristics of colorectal cancer patients by 25-OHD groups in SOCCS (n=2936)

|  | Group 1 (25-OHD < 25nmol/L) | Group 2 (25-OHD 25-50nmol/L) | Group 3 (25-OHD > 50nmol/L) | P |
| --- | --- | --- | --- | --- |
| CRC patients | 868 | 1383 | 685 |  |
| 25-OHD (nmol/L) | 16.08 (6.64) | 35.74 (6.99) | 71.49 (19.96) |  |
| Age of diagnosis (years) | 65.98 (9.28) | 65.61 (9.78) | 66.02 (9.48) | 0.551 |
| Proportion of females | 42.74% | 41.36% | 43.21% | 0.673 |
| AJCC stages |  |  |  | 0.220 |
| I | 145 (16.71%) | 266 (19.23%) | 136 (19.85%) |  |
| II | 316 (36.41%) | 453 (32.75%) | 246 (35.91%) |  |
| III | 304 (35.02%) | 513 (37.09%) | 239 (34.89%) |  |
| IV | 103 (11.87%) | 151 (10.92%) | 64 (9.34%) |  |
| Season of blood collection |  |  |  | 1.65E-07 |
| Fall | 278 (32.03%) | 340 (24.58%) | 176 (25.69%) |  |
| Spring | 196 (22.58%) | 384 (27.77%) | 178 (25.99%) |  |
| Summer | 217 (25%) | 271 (19.6%) | 177 (25.84%) |  |
| Winter | 177 (20.39%) | 388 (28.05%) | 154 (22.48%) |  |
| Cause of death |  |  |  |  |
| CRC | 254 (29.26%) | 299 (21.62%) | 92 (13.43%) | 6.36E-13 |
| All | 386 (44.47%) | 478 (34.56%) | 149 (21.75%) | 1.03E-19 |

The continuous variables were described by using mean and the corresponding standard deviation; the categorical variables were described by using number and proportion.

The P-value was calculated by one-way analysis of variance for continuous variables or χ2 test for categorical variables.

## Table S4 Summarised characteristics of colorectal cancer patients by 25-OHD groups in UKBB (n=3181)

|  | Group 1 (25-OHD < 25nmol/L) | Group 2 (25-OHD 25-50nmol/L) | Group 3 (25-OHD > 50nmol/L) | P |
| --- | --- | --- | --- | --- |
| CRC patients | 399 | 1518 | 1264 |  |
| 25-OHD (nmol/L) | 19.06 (4.99) | 37.68 (7.15) | 65.66 (14.97) |  |
| Age of diagnosis (years) | 64.28 (7.19) | 65.24 (6.47) | 65.64 (6.6) | 0.002 |
| Proportion of females | 44.36% | 41.70% | 39.48% | 0.188 |
| BMI | 29.51 (5.86) | 28.25 (4.61) | 27.26 (4.02) | 4.26E-18 |
| Missing | 24 | 23 | 17 |  |
| Season of blood collection |  |  |  | 0.028 |
| Fall | 110 (27.57%) | 332 (21.87%) | 303 (23.97%) |  |
| Spring | 100 (25.06%) | 426 (28.06%) | 366 (28.96%) |  |
| Summer | 111 (27.82%) | 401 (26.42%) | 353 (27.93%) |  |
| Winter | 78 (19.55%) | 359 (23.65%) | 242 (19.15%) |  |
| Cause of death |  |  |  |  |
| CRC | 112 (28.07%) | 374 (24.64%) | 307 (24.29%) | 0.294 |
| All | 179 (44.86%) | 540 (35.57%) | 445 (35.21%) | 0.001 |

The continuous variables were described by using mean and the corresponding standard deviation; the categorical variables were described by using number and proportion.

The P-value was calculated by one-way analysis of variance for continuous variables or χ2 test for categorical variables.

## Table S5 Schoenfeld residuals for the Cox regression models before and after categorising data into subgroups in SOCCS

|  | All | | | AJCC1 | | | AJCC2 | | | AJCC3 | | | AJCC4 | | |
| --- | --- | --- | --- | --- | --- | --- | --- | --- | --- | --- | --- | --- | --- | --- | --- |
| Variable | chisq | df | p | chisq | df | p | chisq | df | p | chisq | df | p | chisq | df | p |
| **Cancer-specific death** | | | | | | | | | | | | | | | |
| Vitamin D | 3.417 | 1 | 0.07 | 0.21 | 1 | 0.65 | 0.84 | 1 | 0.36 | 3.73 | 1 | 0.05 | 0.01 | 1 | 0.93 |
| Age at diagnosis | 2.315 | 1 | 0.13 | 0.06 | 1 | 0.80 | 0.39 | 1 | 0.53 | 0.05 | 1 | 0.82 | 0.01 | 1 | 0.91 |
| Sex | 2.573 | 1 | 0.11 | 0.18 | 1 | 0.67 | 0.95 | 1 | 0.33 | 0.48 | 1 | 0.49 | 2.49 | 1 | 0.11 |
| Tumour site | 3.821 | 2 | 0.15 | 0.03 | 2 | 0.99 | 2.12 | 2 | 0.35 | 0.46 | 2 | 0.80 | 0.02 | 2 | 0.99 |
| Sampling season | 0.844 | 3 | 0.84 | 1.93 | 3 | 0.59 | 1.33 | 3 | 0.72 | 2.91 | 3 | 0.41 | 5.17 | 3 | 0.16 |
| AJCC | 74.64 | 1 | <2e-16 | / | / | / | / | / | / | / | / | / | / | / | / |
| Global | 82.011 | 9 | 6.40E-14 | 2.27 | 8 | 0.97 | 5.45 | 8 | 0.71 | 7.36 | 8 | 0.50 | 7.79 | 8 | 0.45 |
| **All-cause death** | | | | | | | | | | | | | | | |
| Vitamin D | 10.88 | 1 | 0.001 | 0.01 | 1 | 0.94 | 0.80 | 1 | 0.37 | 8.45 | 1 | 0.004 | 0.18 | 1 | 0.67 |
| Age at diagnosis | 51.44 | 1 | 7.40E-13 | 9.94 | 1 | 0.002 | 0.92 | 1 | 0.34 | 10.48 | 1 | 0.001 | 0.15 | 1 | 0.70 |
| Sex | 0.07 | 1 | 0.78 | 0.91 | 1 | 0.34 | 0.00 | 1 | 0.97 | 0.52 | 1 | 0.47 | 1.48 | 1 | 0.22 |
| Tumour site | 2.68 | 2 | 0.26 | 0.28 | 2 | 0.87 | 2.64 | 2 | 0.27 | 0.02 | 2 | 0.99 | 0.08 | 2 | 0.96 |
| Sampling season | 0.37 | 3 | 0.95 | 2.26 | 3 | 0.52 | 1.02 | 3 | 0.8 | 2.34 | 3 | 0.50 | 3.36 | 3 | 0.34 |
| AJCC | 157.04 | 1 | <2e-16 | / | / | / | / | / | / | / | / | / | / | / | / |
| Global | 207.40 | 9 | <2e-16 | 14.75 | 8 | 0.06 | 5.89 | 8 | 0.66 | 19.94 | 8 | 0.01 | 5.37 | 8 | 0.72 |

For the Cox regression model of SOCCS, based on results from Schoenfeld residuals, the AJCC stage is non-proportionally associated with CRC survival. We then re-estimate the Schoenfeld residuals after categorising the data based on AJCC stages.

## Table S6 Schoenfeld residuals for the Cox regression model before and after categorising data into subgroups in UKBB

|  | **Colorectal cancer** | | | **Colon cancer** | | | **Rectal cancer** | | |
| --- | --- | --- | --- | --- | --- | --- | --- | --- | --- |
| Variable | Chi-square | df | P | Chi-square | df | P | Chi-square | df | P |
| **Cancer-specific death** | | | | | | | | | |
| Vitamin D | 2.41 | 2 | 0.30 | 2.59 | 2 | 0.27 | 0.72 | 2 | 0.7 |
| Age at diagnosis | 0.70 | 1 | 0.40 | 0.92 | 1 | 0.34 | 0.12 | 1 | 0.73 |
| Sex | 4.34 | 1 | 0.04 | 2.45 | 1 | 0.12 | 0.10 | 1 | 0.75 |
| Tumour site | 13.44 | 2 | 0.0012 | / | / | / | / | / | / |
| Sampling season | 6.64 | 3 | 0.08 | 4.54 | 3 | 0.21 | 4.97 | 3 | 0.17 |
| BMI | 0.03 | 1 | 0.85 | 0.08 | 1 | 0.78 | 0.37 | 1 | 0.54 |
| Global | 26.46 | 10 | 0.0032 | 11.03 | 8 | 0.2 | 6.74 | 8 | 0.57 |
| **All-cause death** | | | | | | | | | |
| Vitamin D | 0.72 | 2 | 0.70 | 0.78 | 2 | 0.68 | 1.37 | 2 | 0.50 |
| Age at diagnosis | 1.47 | 1 | 0.23 | 0.17 | 1 | 0.68 | 4.44 | 1 | 0.04 |
| Sex | 2.62 | 1 | 0.11 | 2.04 | 1 | 0.15 | 0.13 | 1 | 0.71 |
| Tumour site | 15.65 | 2 | 0.0004 | / | / | / | / | / | / |
| Sampling season | 4.80 | 3 | 0.19 | 1.49 | 3 | 0.69 | 7.34 | 3 | 0.06 |
| BMI | 0.09 | 1 | 0.76 | 0.00 | 1 | 0.99 | 0.09 | 1 | 0.77 |
| Global | 24.52 | 10 | 0.01 | 4.17 | 8 | 0.84 | 14.25 | 8 | 0.08 |

For the Cox regression models of UKBB, based on results from Schoenfeld residuals, the tumour site is non-proportional related to the survival of CRC. We then re-estimate the Schoenfeld residuals after categorising the data based on the tumour site (colon & rectum).

## Table S7 Summarised characteristics of colorectal cancer patients by subgroups

|  | CRC cases number | CRC specific death | All death | Vitamin D level | Follow-up year |
| --- | --- | --- | --- | --- | --- |
| SOCCS |  |  |  |  |  |
| AJCC stage I | 663 (19.38%) | 39 (7.13%) | 139 (25.41%) | 34.43 (24.04, 49.77) | 7.26 (4.16, 13.47) |
| AJCC stage II | 1174 (34.32%) | 148 (14.58%) | 294 (28.97%) | 33.00 (22.91, 49.07) | 6.23 (3.48, 13.43) |
| AJCC stage III | 1226 (35.84%) | 269 (25.47%) | 376 (35.61%) | 34.20 (23.04, 49.99) | 4.62 (2.76, 10.17) |
| AJCC stage IV | 358 (10.46%) | 189 (59.43%) | 204 (64.15%) | 31.06 (21.55, 47.16) | 2.26 (1.15, 4.07) |
| Tumour site-colon | 1788 (60.90%) | 394 (22.04%) | 607 (33.95%) | 33.33 (22.90, 48.98) | 5.06 (2.79, 11.90) |
| Tumour site-rectum | 1091 (37.16%) | 241 (22.09%) | 375 (34.37%) | 33.31 (23.55, 48.37) | 5.49 (3.04, 12.29) |
| rs11568820-GG | 1836 (62.53%) | 395 (21.51%) | 622 (33.88%) | 32.75 (23.02, 48.20) | 5.24 (2.86, 12.07) |
| rs11568820-AA/AG | 1100 (37.46%) | 250 (22.73%) | 391 (35.55%) | 34.74 (23.00, 49.22) | 5.27 (2.93, 12.43) |
| UKBB |  |  |  |  |  |
| Tumour site-colon | 3299 (62.59%) | 534 (25.86%) | 785 (38.01%) | 46.50 (31.90, 61.30) | 6.42 (2.81, 8.87) |
| Tumour site-rectum | 1972 (37.41%) | 252 (23.18%) | 368 (33.85%) | 49.40 (34.70, 64.95) | 6.43 (4.28, 8.93) |
| rs11568820-GG | 1964 (61.74%) | 487 (24.80%) | 727 (37.02%) | 44.39 (31.68, 59.41) | 6.37 (3.43, 8.85) |
| rs11568820-AA/AG | 1181 (37.13%) | 292 (24.72%) | 420 (35.56%) | 44.90 (31.71, 56.40) | 6.50 (3.29, 8.98) |

Continuous variables are presented with median and interquartile range.

## Table S8 Observational analyses for the association between 25-OHD and colorectal cancer survival in SOCCS and UKBB by stages and tumour sites

|  | CRC death | Overall death |
| --- | --- | --- |
|  | **SOCCS (n=2936)** | |
| **AJCC=1** |  |  |
| HR (95%CI) | 0.88 (0.63, 1.23) | 0.96 (0.80, 1.15) |
| P | 0.46 | 0.64 |
| **AJCC=2** |  |  |
| HR (95%CI) | 0.76 (0.65, 0.90) | 0.78 (0.69, 0.88) |
| P | 0.002 | 4.79e-05 |
| **AJCC=3** |  |  |
| HR (95%CI) | 0.74 (0.66, 0.83) | 0.80 (0.72, 0.89) |
| P | 5.75e-07 | 1.78e-05 |
| **AJCC=4** |  |  |
| HR (95%CI) | 0.84 (0.73, 0.97) | 0.84 (0.73, 0.97) |
| P | 0.020 | 0.020 |
| **Tumour site-colon** |  |  |
| HR (95%CI) | 0.76 (0.69, 0.84) | 0.81 (0.74, 0.88) |
| P | 6.82e-08 | 2.87e-07 |
| **Tumour site-rectum** |  |  |
| HR (95%CI) | 0.78 (0.68, 0.89) | 0.82 (0.73, 0.91) |
| P | 0.0002 | 0.0002 |
|  | **UKBB (n=3181)** | |
| **Tumour site-colon** |  |  |
| HR (95%CI) | 0.94 (0.87, 1.04) | 0.95 (0.88, 1.02) |
| P | 0.237 | 0.022 |
| **Tumour site-rectum** |  |  |
| HR (95%CI) | 0.92 (0.81, 1.04) | 0.85 (0.77, 0.94) |
| P | 0.187 | 0.002 |

CRC: colorectal cancer, the Bonferroni corrected P value threshold equals 0.004, Age and sex were adjusted. These Cox regression models were generated based on 25-OHD as a continuous variable. The HR was estimated based on per unit change of rank-based inverse-normal transformed May-adjusted circulating 25-OHD

## Table S9 Observational analyses for the association between 25-OHD and colorectal cancer survival in SOCCS and UKBB by rs11568820 genotypes

|  | Group 1* | Group 2* | | | Group 3* | | |  |  |  |
| --- | --- | --- | --- | --- | --- | --- | --- | --- | --- | --- |
|  |  | HR | 95%CI | P | HR | 95%CI | P | P trend | P interaction | Number of patients |
| CRC death-UKBB |  |  |  |  |  |  |  |  |  |  |
| GG^#^ | Ref | 0.79 | 0.61 to 1.03 | 0.086 | 0.76 | 0.58 to 1 | 0.048 | 0.055 | 0.690 | 1964 |
| AA/AG^#^ | Ref | 0.90 | 0.63 to 1.29 | 0.563 | 0.88 | 0.61 to 1.27 | 0.506 | 0.390 |  | 1217 |
|  |  |  |  |  |  |  |  |  |  |  |
| All cause of death-UKBB |  |  |  |  |  |  |  |  |  |  |
| GG^#^ | Ref | 0.75 | 0.60 to 0.93 | 0.009 | 0.75 | 0.6 to 0.93 | 0.009 | 0.038 | 0.312 | 1964 |
| AA/AG^#^ | Ref | 0.70 | 0.53 to 0.92 | 0.010 | 0.64 | 0.48 to 0.85 | 0.002 | 0.011 |  | 1217 |
|  |  |  |  |  |  |  |  |  |  |  |
| CRC death-SOCCS |  |  |  |  |  |  |  |  |  |  |
| GG^#^ | Ref | 0.64 | 0.52 to 0.79 | 3.93E-05 | 0.41 | 0.3 to 0.56 | 3.87E-08 | 3.19e-11 | 0.014 | 1836 |
| AA/AG^#^ | Ref | 1.03 | 0.78 to 1.37 | 0.811 | 0.70 | 0.48 to 1.01 | 0.056 | 0.033 |  | 1100 |
|  |  |  |  |  |  |  |  |  |  |  |
| All cause of death-SOCCS |  |  |  |  |  |  |  |  |  |  |
| GG^#^ | Ref | 0.75 | 0.63 to 0.88 | 6.28E-04 | 0.49 | 0.38 to 0.63 | 4.48E-08 | 5.03e-11 | 0.008 | 1836 |
| AA/AG^#^ | Ref | 1.06 | 0.84 to 1.32 | 0.637 | 0.81 | 0.6 to 1.08 | 0.148 | 1.06 |  | 1100 |

SOCCS: Study of Colorectal Cancer in Scotland, UKBB: UK Biobank, HR: hazard ratio, CI: confidence interval.

*: Group 1 < 25 nmol/L, Group 2 = 25-50 nmol/L, Group 3 > 50 nmol/L; #: rs11568820 genotypes.

Model: Adjusted for age and sex; P trend was tested based on continuous rank-based inverse-normal transformed 25-OHD; P interaction: the P value of the interaction term in the model based on continuous rank-based inverse-normal transformed 25-OHD

## Table S10 Summarised characteristics of colorectal cancer patients in the Mendelian randomisation study

|  | SOCCS | UKBB |
| --- | --- | --- |
| CRC patients | 5675 | 5847 |
| PRS | -0.41 (0.20) | -0.39 (0.21) |
| Age of diagnosis (years) | 63.22 (11.42) | 61.47 (8.57) |
| Proportion of females | 2441 (43.01%) | 2488 (42.55%) |
| Cause of death |  |  |
| CRC | 1358 (23.93%) | 1062 (1816%) |
| All | 1918 (33.80%) | 1759 (30.08%) |

The continuous variables were described by using mean and the corresponding standard deviation; the categorical variables were described by using number and proportion.

## Table S11 Performance of 25-OHD polygenic risk score in SOCCS and UK Biobank

| Correlation coefficient (P-value) | | | Correlation coefficient (P-value) | | | |
| --- | --- | --- | --- | --- | --- | --- |
| UKBB | | | SOCCS | | | |
| PRS | | | PRS | | | |
| Total (n=443383) | CRC cases (n=3487) | Non-CRC cases (n=438,105) | Total (n=5839) | CRC cases (n=3421) | Non-CRC cases (n=2418) |  |
| 0.21 (0) | 0.20 (9.42e-31) | 0.21 (0) | 0.10 (2.40e-13) | 0.10 (1.60e-8) | 0.10 (2.61e-6) |  |
| PRS_6_ | | | PRS_6_ | | | |
| Total (n=443383) | CRC cases (n=3487) | Non-CRC cases (n=438,105) | Total (n=5839) | CRC cases (n=3421) | Non-CRC cases (n=2418) |  |
| 0.17 (0) | 0.16 (1.07e-18) | 0.17 (0) | 0.08 (9.66e-11) | 0.08 (7.24e-6) | 0.10 (1.64e-6) |  |

PRS: The PRS generated from the new 25-OHD GWAS in the UK Biobank. PRS_6_: The PRS generated from the SUNLIGHT 25-OHD GWAS.

## Table S12 Power estimation

| Power | UKBB (n=5847) | | SOCCS (n=5675) | | | UKBB + SOCCS (n=11522) | |
| --- | --- | --- | --- | --- | --- | --- | --- |
|  | CRC specific death (n=1062) | All death (n=1759) | CRC specific death (n=1358) | All death (n=1918) | CRC specific death (n=2420) | | All death (n=3677) |
|  | R^2^=10.5%, F statistic=685.96 | | R^2^=10.5%, F statistic=665.55 | | | R^2^=10.5%, F statistic=1351.51 | |
| 80% | HR =0.72 or  HR =1.39 | HR =0.77 or  HR =1.30 | HR =0.75 or  HR =1.33 | HR =0.78 or  HR =1.28 | HR =0.81 or  HR =1.23 | | HR =0.84 or  HR =1.19 |
|  | R^2^=5.7%, F statistic=353.30 | | R^2^=5.7%, F statistic=342.91 | | | R^2^=5.7%, F statistic=696.33 | |
| 80% | HR =0.63 or  HR =1.59 | HR =0.71 or  HR =1.41 | HR =0.67 or  HR =1.49 | HR =0.71 or  HR =1.41 | HR =0.75 or  HR =1.33 | | HR =0.78 or  HR =1.28 |

F statistics were estimated based on the formula: $F=R^{2}\times(N-2)/(1-R^{2})$

HR: hazard ratio, SOCCS: Study of Colorectal Cancer in Scotland, UKBB: UK Biobank, CRC: colorectal cancer.

## Table S13 Results of individual-level MR analyses for effect of rank-based inverse-normal transformed circulating 25-OHD on colorectal cancer survival in SOCCS and UKBB

|  | Effect matrix | SOCCS (n=5675)  Covariates: age, sex and AJCC | | SOCCS (n=5675)  Covariates: age and sex | | UK Biobank (n=5847)  Covariates: age and sex | | | Meta-analysis (n=11,522)  Covariates: age and sex | | |
| --- | --- | --- | --- | --- | --- | --- | --- | --- | --- | --- | --- |
|  |  | CRC death  (n=1358) | Overall death  (n=1918) | CRC death  (n=1358) | Overall death  (n=1918) | CRC death  (n=1062) | Overall death  (n=1759) | CRC death  (n=2420, I^2^=0%, P_het_=0.59) | | Overall death  (n=3677, I^2^=0%, P_het_=0.35) |  |
| PRS | HR (95%CI) | 1.18 (0.90, 1.55) | 1.24 (0.99, 1.56) | 1.03 (0.79, 1.34) | 1.15 (0.92, 1.44) | 0.92 (0.69, 1.24) | 0.99 (0.79, 1.24) | 0.98 (0.80, 1.19) | | 1.07 (0.91, 1.25) |  |
|  | P | 0.24 | 0.06 | 0.84 | 0.22 | 0.59 | 0.91 | 0.83 | | 0.43 |  |

For UKBB, the number of SNPs to generate the PRS was 113; for SOCCS, the number of SNPs to generate the PRS was 107.

## Table S14 Results of summary-level MR analyses for effect of rank-based inverse-normal transformed circulating 25-OHD on colorectal cancer survival in SOCCS and UKBB

|  | Effect matrix | SOCCS (n=5675)  Covariates: age, sex and AJCC | | UK Biobank (n=5847)  Covariates: age and sex | | | Meta-analysis (n=11,522) | |
| --- | --- | --- | --- | --- | --- | --- | --- | --- |
|  |  | CRC death  (n=1358) | Overall death  (n=1918) | CRC death  (n=1062) | Overall death  (n=1759) | CRC death  (n=2420, I^2^=22%, P_het_=0.26) | | Overall death  (n=3677, I^2^=46%, P_het_=0.18) |
| Inverse variance weighed | HR (95%CI) | 1.17 (0.88,1.57) | 1.24 (0.98, 1.58) | 0.92 (0.68, 1.24) | 0.98 (0.77, 1.25) | 1.04 (0.85, 1.28) | | 1.10 (0.93, 1.31) |
|  | P | 0.27 | 0.08 | 0.57 | 0.89 | 0.70 | | 0.25 |
| Median-based | HR (95%CI) | 1.29 (0.85, 1.96) | 1.14 (0.80, 1.61) | 0.78 (0.50, 1.22) | 0.77 (0.54, 1.10) | / | | / |
|  | P | 0.23 | 0.47 | 0.20 | 0.15 | / | | / |
| MR-EGGER | HR (95%CI) | 1.22 (0.82, 1.82) | 1.19 (0.85, 1.65) | 0.81 (0.53, 1.23) | 0.91 (0.65, 1.28) | / | | / |
|  | P | 0.32 | 0.31 | 0.32 | 0.59 | / | | / |
| MR-PRESSO | HR (95%CI) | 1.17 (0.88,1.57) | 1.24 (0.98, 1.58) | 0.92 (0.68, 1.24) | 0.98 (0.77, 1.25) | / | | / |
|  | P | 0.27 | 0.08 | 0.57 | 0.89 | / | | / |
| MR-RAPS | HR (95%CI) | 1.19 (0.89, 1.60) | 1.24 (0.98, 1.58) | 0.90 (0.67, 1.23) | 0.95 (0.74, 1.22) | / | | / |
|  | P | 0.24 | 0.08 | 0.54 | 0.70 | / | | / |
| MR contamination mixture | HR (95%CI) | 1.21 (0.90, 1.68) | 1.21 (0.91, 1.67) | 0.91 (0.71, 1.20) | 0.86 (0.60, 1.20) | / | | / |
|  | P | 0.22 | 0.15 | 0.50 | 0.38 | / | | / |
|  | P_het_ | 0.32 | 0.38 | 0.40 | 0.27 | / | | / |
|  | P_int_ | 0.77 | 0.69 | 0.39 | 0.53 | / | | / |

For UKBB, the number of vitamin D genetic variants was 113; for SOCCS, the number of vitamin D genetic variants was 107.

## Table S15 Results of individual-level MR analyses by taking rs11568820 or PRS after categorising based on rs11568820 as genetic instrument to colorectal cancer survival in SOCCS and UKBB

|  | Effect matrix | SOCCS (n=5675)  Covariates: age, sex and AJCC | | SOCCS (n=5675)  Covariates: age and sex | | UK Biobank (n=5847)  Covariates: age and sex | |
| --- | --- | --- | --- | --- | --- | --- | --- |
|  |  | CRC death  (n=1358) | Overall death  (n=1918) | CRC death  (n=1358) | Overall death  (n=1918) | CRC death  (n=1062) | Overall death  (n=1759) |
| rs11568820 | HR (95%CI) | 1.02 (0.93, 1.12) | 1.01 (0.94, 1.09) | 1.01 (0.92, 1.11) | 1.00 (0.93, 1.09) | 0.97 (0.87, 1.07) | 1.03 (0.95, 1.12) |
|  | P | 0.675 | 0.739 | 0.829 | 0.896 | 0.550 | 0.471 |
| PRS (rs11568820: GG); N=3501 in SOCCS; N=3586 UKBB | HR (95%CI) | 1.33 (0.94, 1.90) | 1.32 (0.98, 1.77) | 1.12 (0.79, 1.57) | 1.21 (0.91, 1.61) | 0.83 (0.57, 1.20) | 0.97 (0.73, 1.29) |
|  | P | 0.11 | 0.06 | 0.53 | 0.19 | 0.33 | 0.85 |
| PRS (rs11568820: AA/AG); N=2151 in SOCCS, N=2261 in UKBB | HR (95%CI) | 0.99 (0.64, 1.51) | 1.12 (0.78, 1.60) | 0.88 (0.57, 1.36) | 1.03 (0.72, 1.49) | 1.10 (0.68, 1.77) | 1.02 (0.70, 1.48) |
|  | P | 0.95 | 0.55 | 0.57 | 0.86 | 0.70 | 0.93 |
|  | P_interaction_ | 0.30 | 0.47 | 0.41 | 0.50 | 0.35 | 0.81 |

For UKBB, the number of SNPs to generate the PRS was 113; for SOCCS, the number of SNPs to generate the PRS was 107; P_interaction_, P-value of the interaction term from the main model.

## Table S16 MR results between vitamin D and CRC survival using six vitamin D related loci detected by SUNLIGHT GWAS

|  | Effect matrix | SOCCS (n=5675)  Covariates: age, sex and AJCC | | SOCCS (n=5675)  Covariates: age and sex | | UK Biobank (n=3487)  Covariates: age and sex | |  |
| --- | --- | --- | --- | --- | --- | --- | --- | --- |
|  |  | CRC death  (n=1358) | Overall death  (n=1918) | CRC death  (n=1358) | Overall death  (n=1918) | CRC death  (n=878) | Overall death  (n=1278) | |
| PRS_6_ | HR (95%CI) | 1.11 (0.78, 1.58) | 1.10 (0.82, 1.48) | 1.01 (0.71, 1.42) | 1.07 (0.80, 1.43) | 0.75 (0.50, 1.14) | 0.82 (0.58, 1.15) | |
|  | P | 0.55 | 0.51 | 0.98 | 0.65 | 0.18 | 0.25 | |

## Figure S1 Flow chart of Vitamin D genetic variants selection

GWAS: genome-wide association study, MAF: minor allele frequency, LD: linkage disequilibrium.


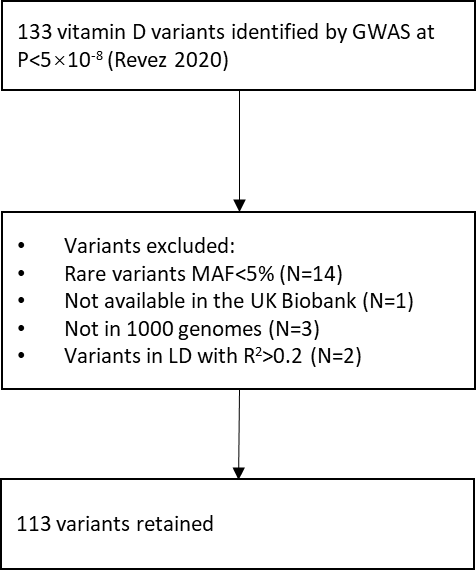


## Figure S2 Predicted hazard ratio by May-standardised 25-OHD in SOCCS and UKBB


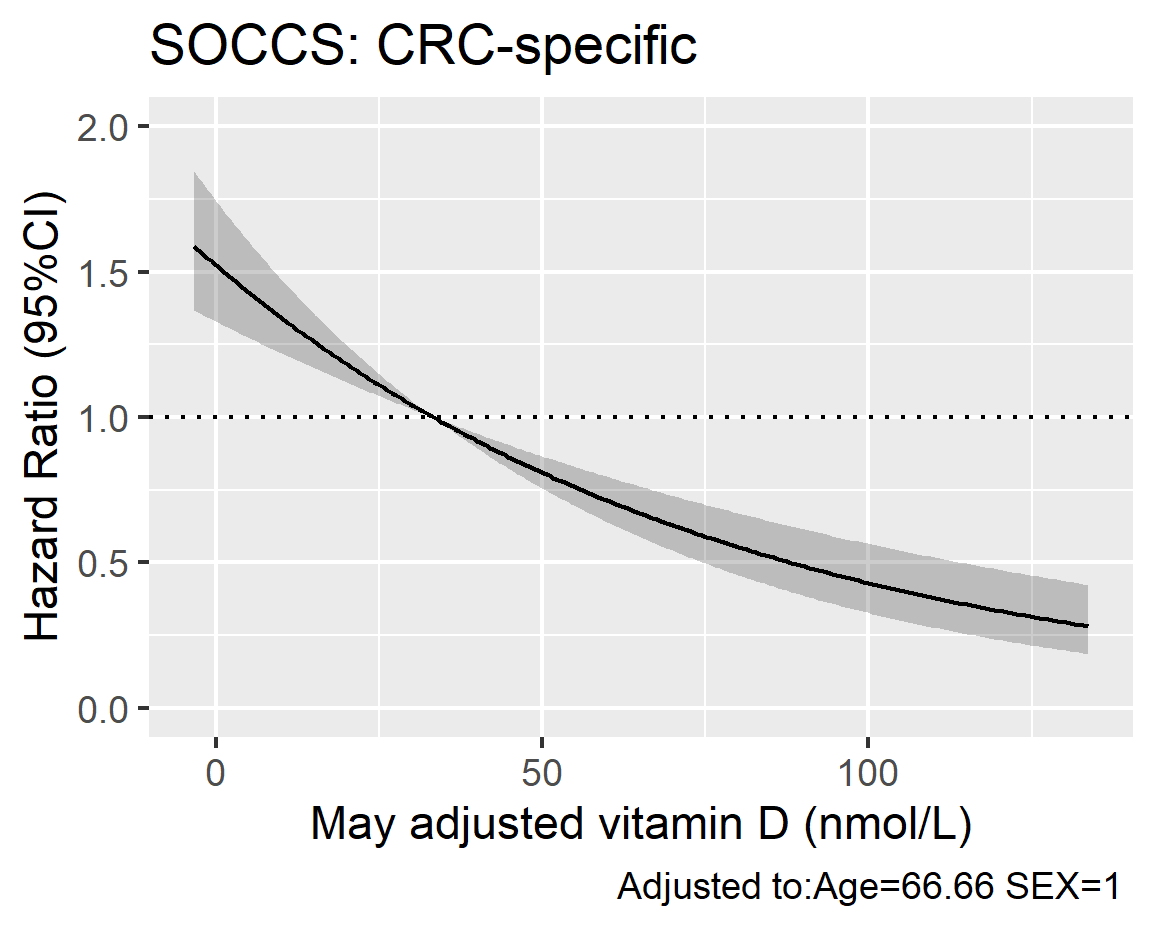

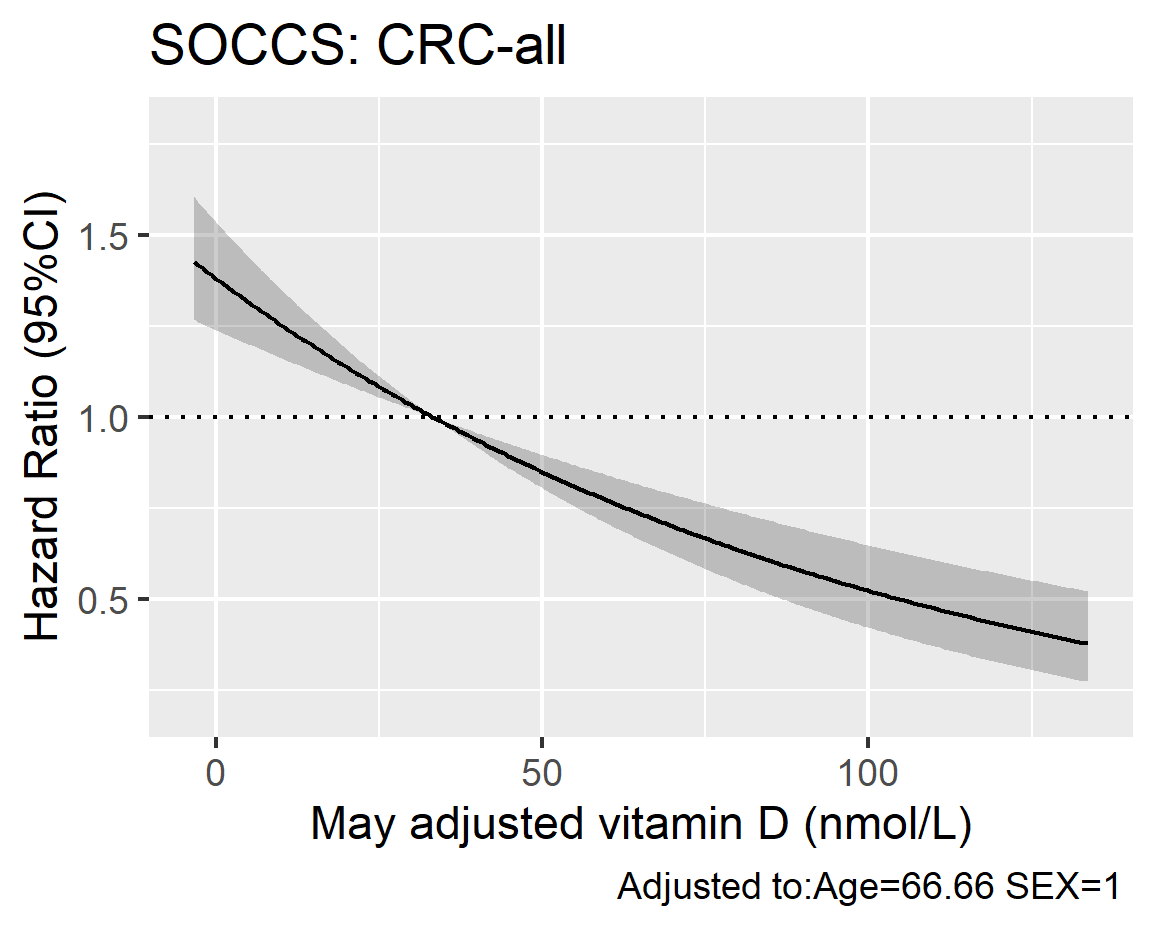


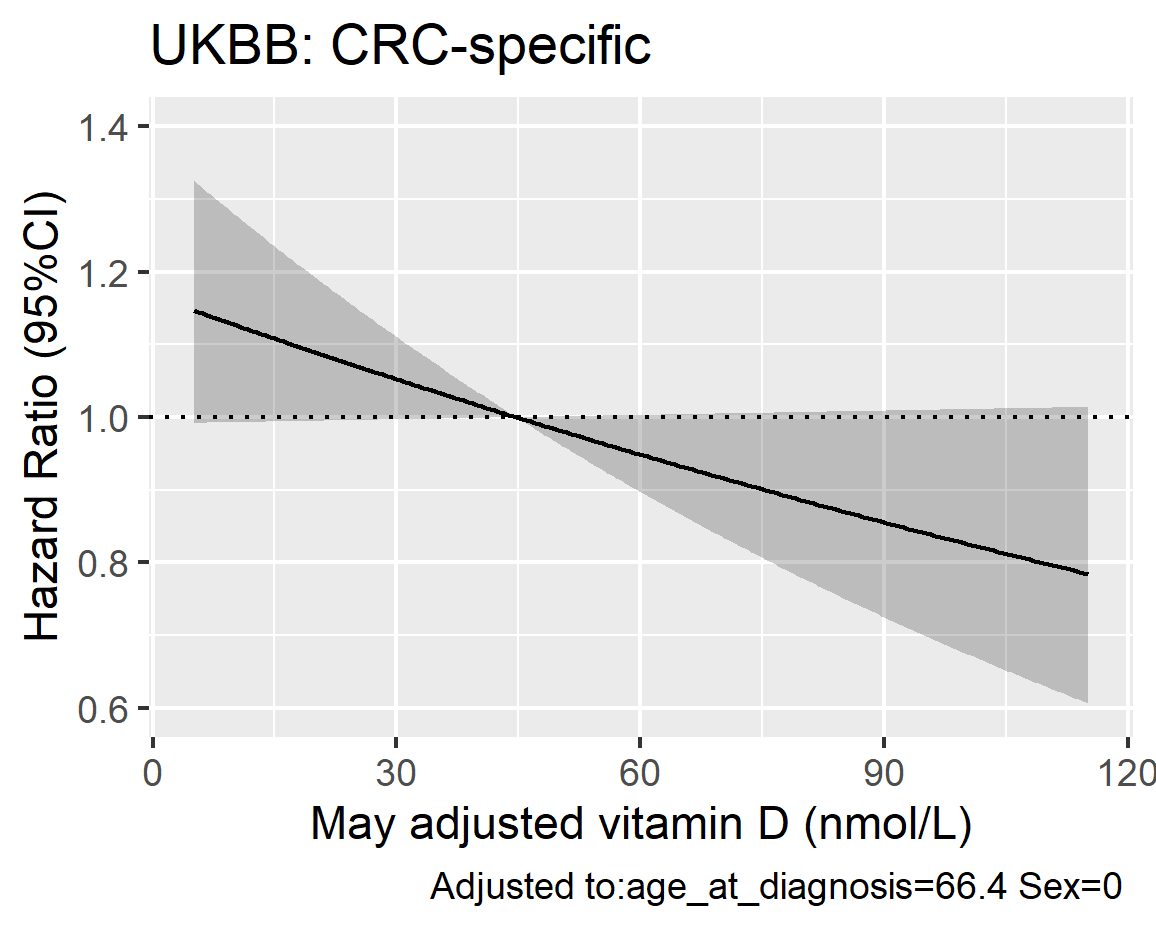

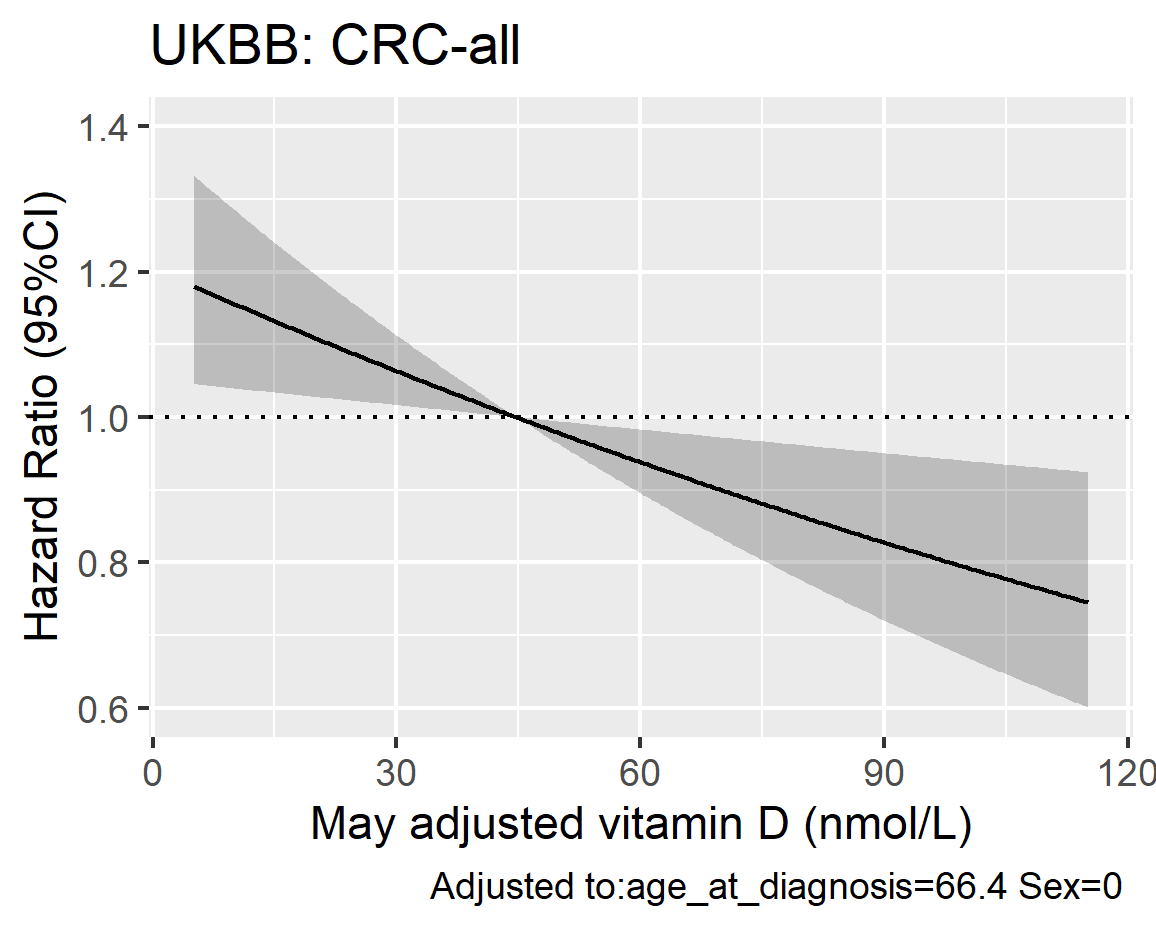


## Figure S3 Kaplan-Meier survival estimates of colorectal cancer-specific (A: SOCCS; C: UKBB) and overall survival (B: SOCCS; D: UKBB) among incident and prevalent cases

**A: Survival estimate in SOCCS (CRC-specific) B: Survival estimate in SOCCS (overall)**


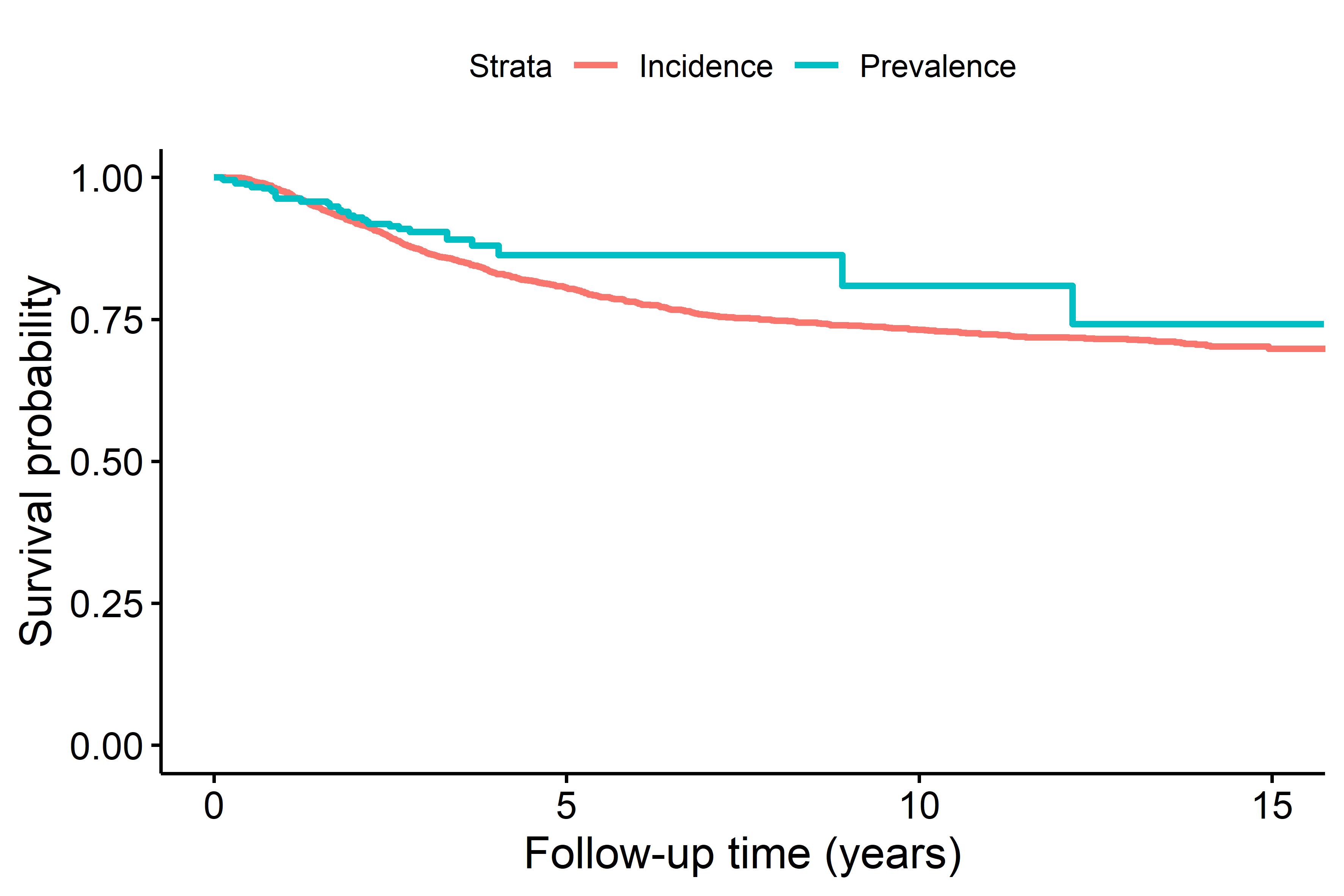

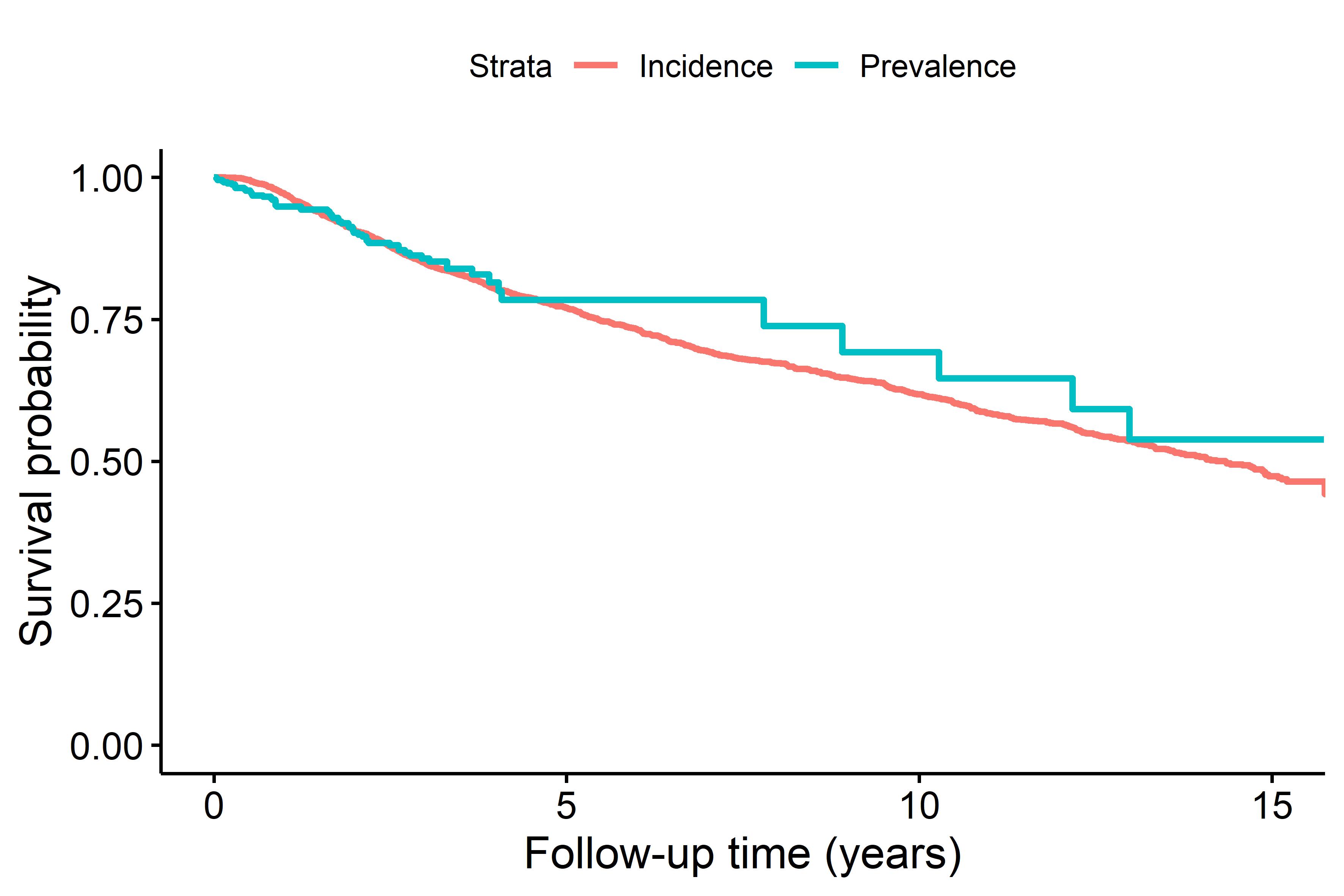


**C: Survival estimate in UKBB (CRC-specific) D: Survival estimate in UKBB (overall)**

**
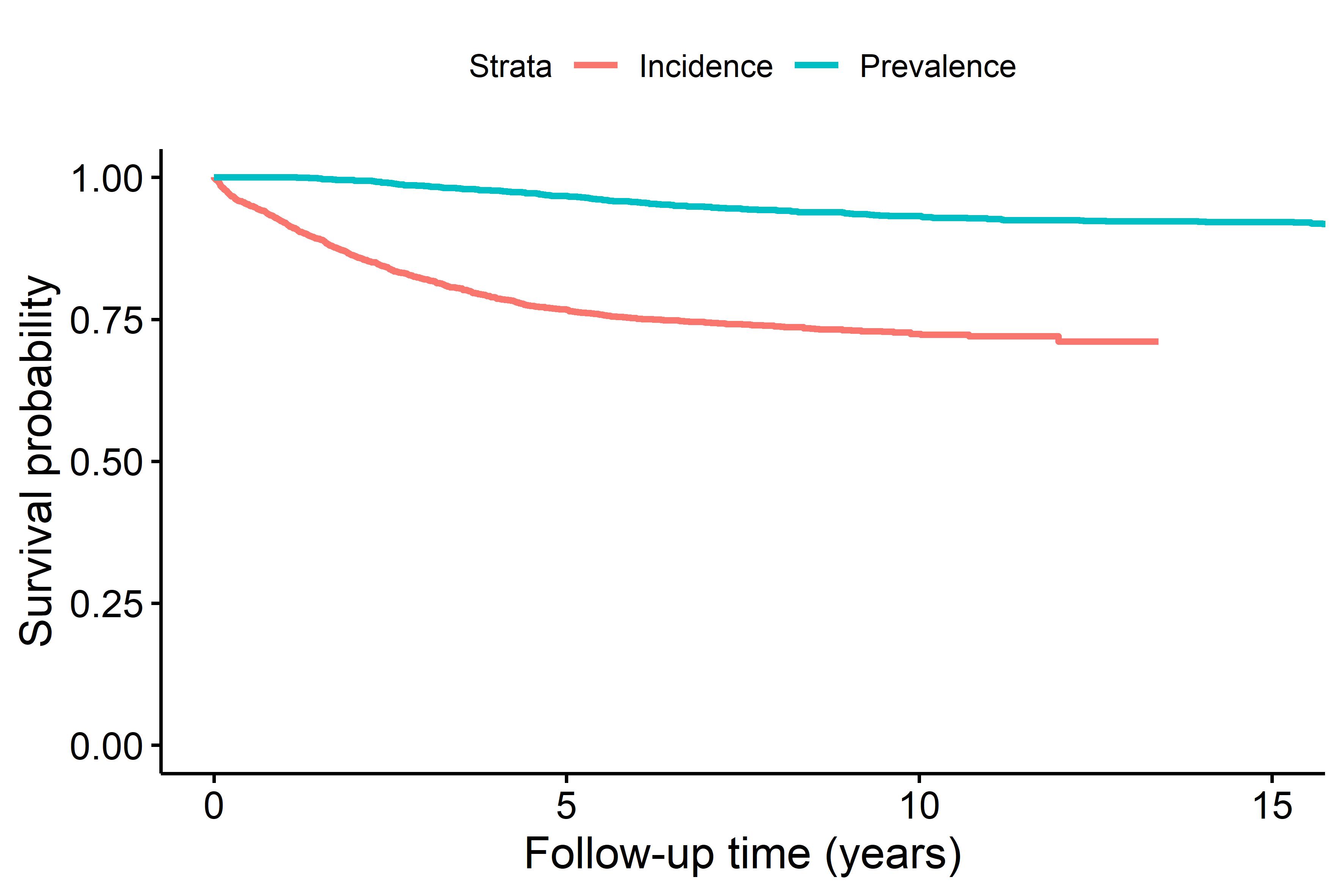

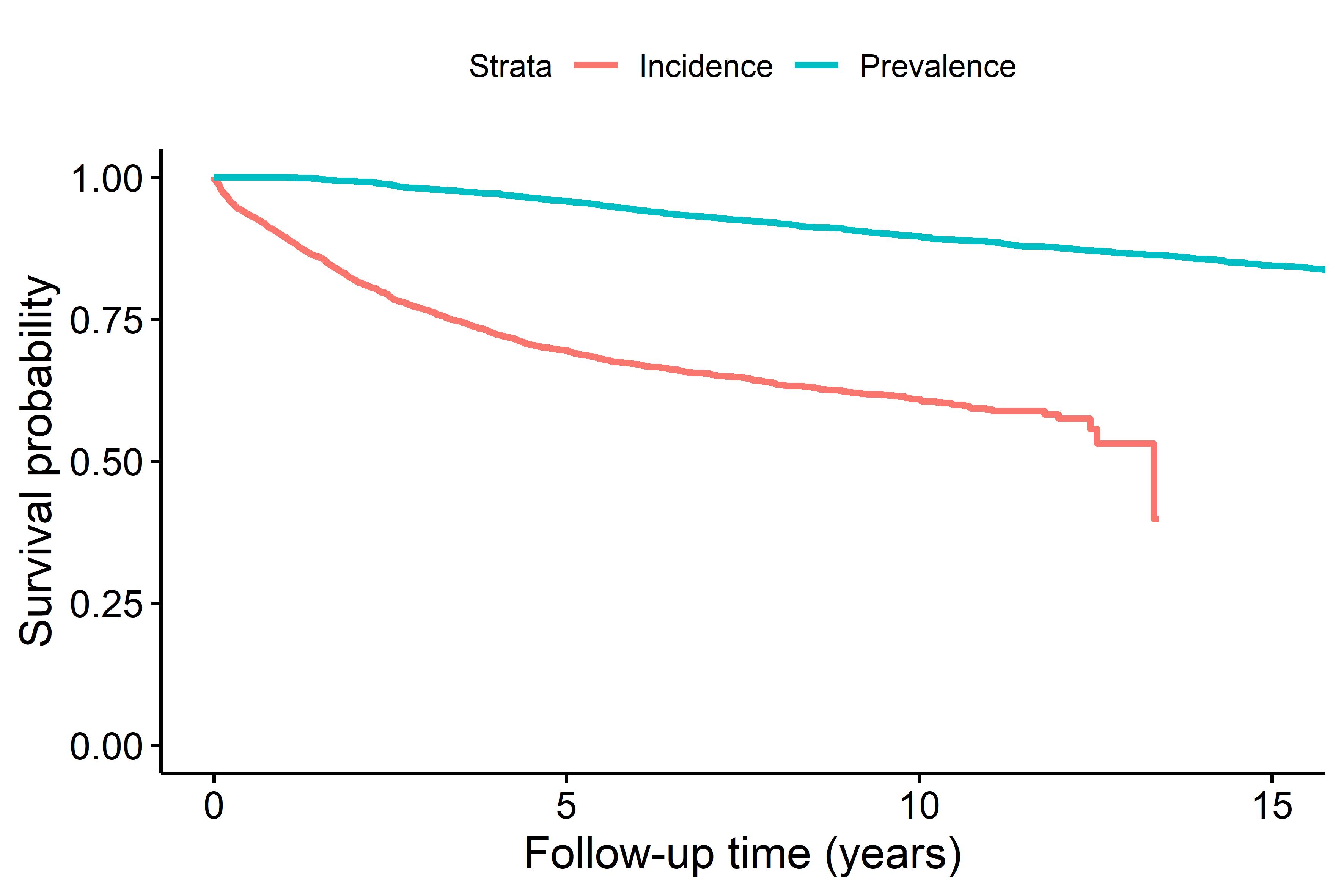
**

## Figure S4 Scatter plots for summary-level Mendelian randomisation analyses

**A: SOCCS (CRC-specific) B: SOCCS (all-cause)**


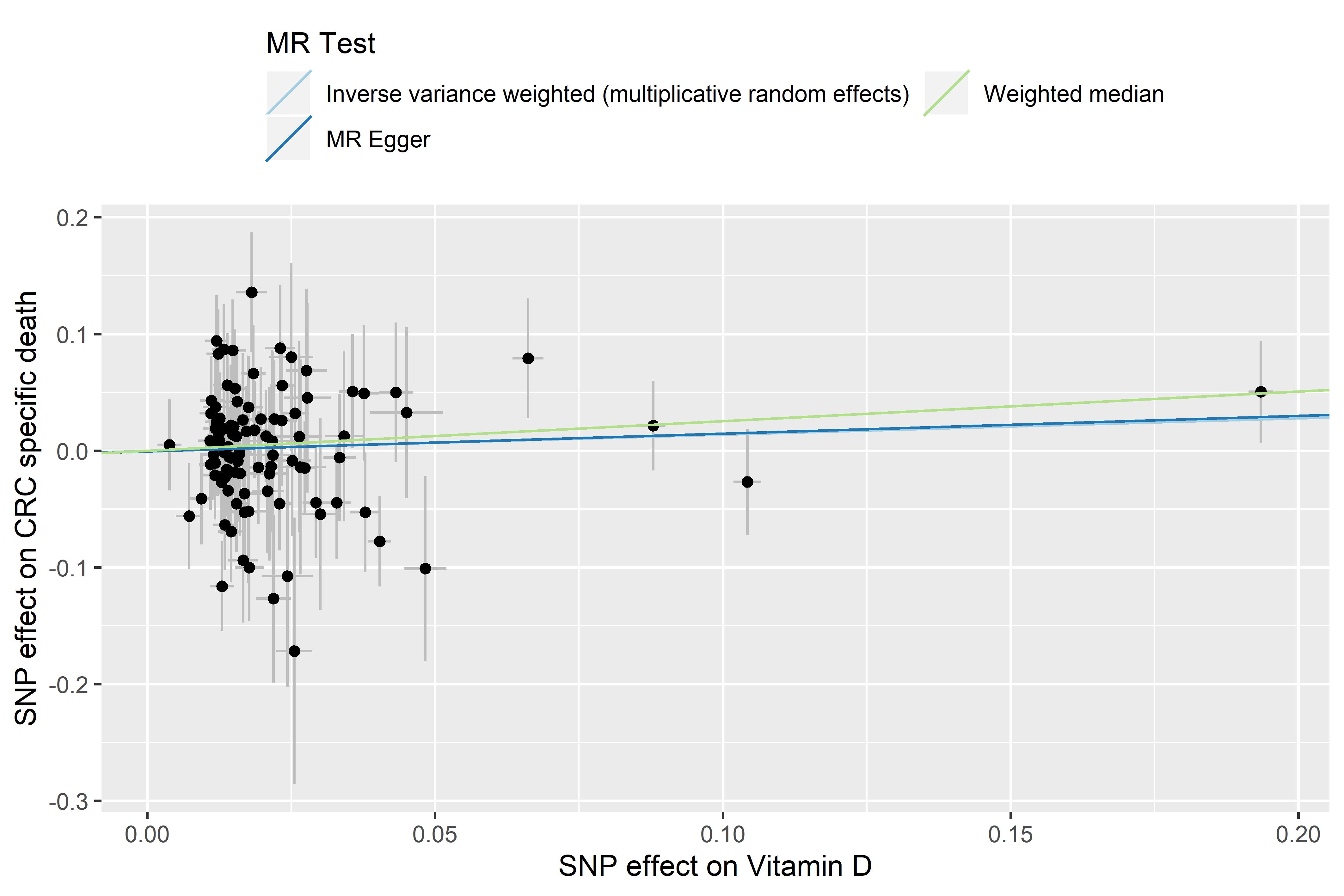

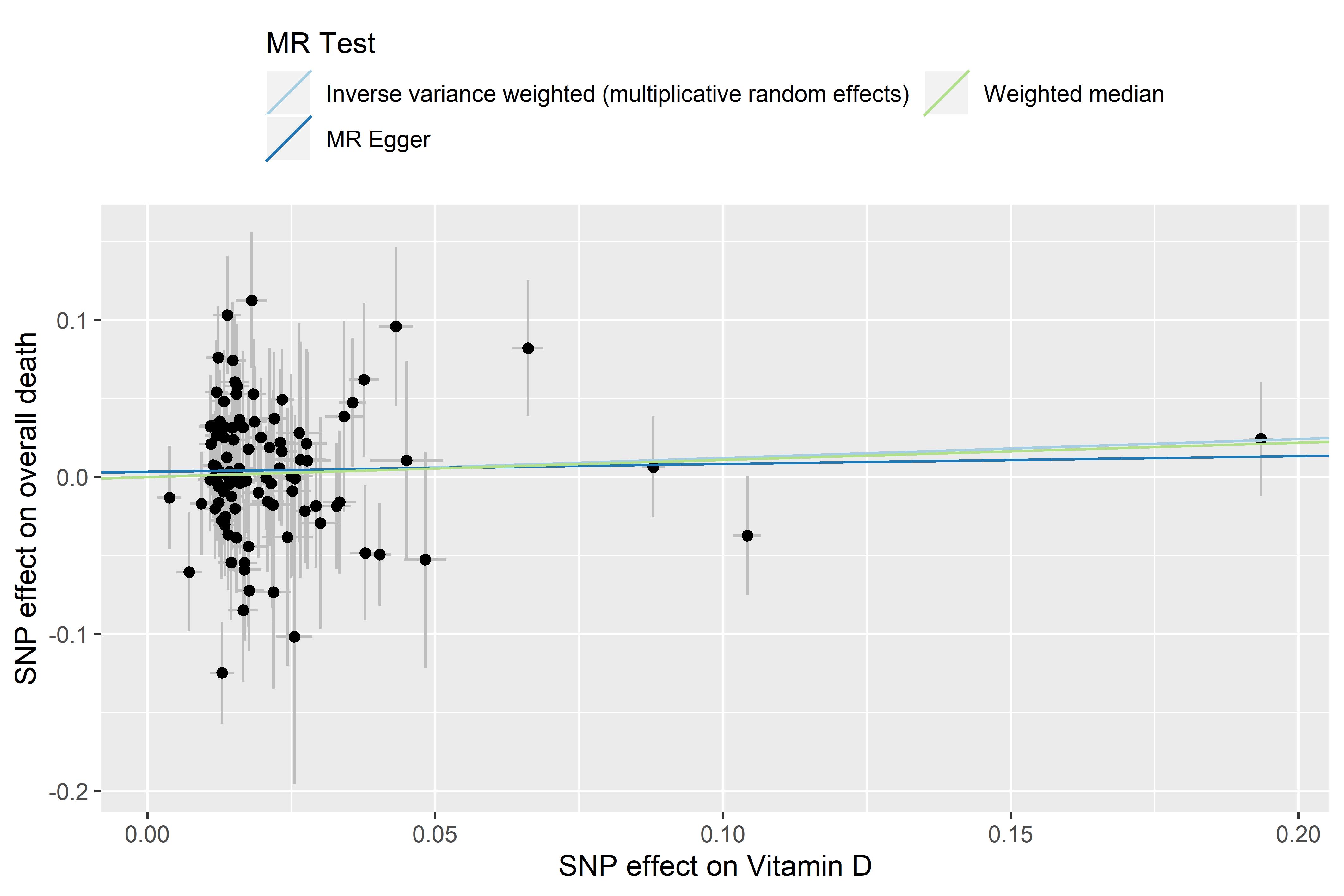


**A: UKBB (CRC-specific) B: UKBB (all-cause)**


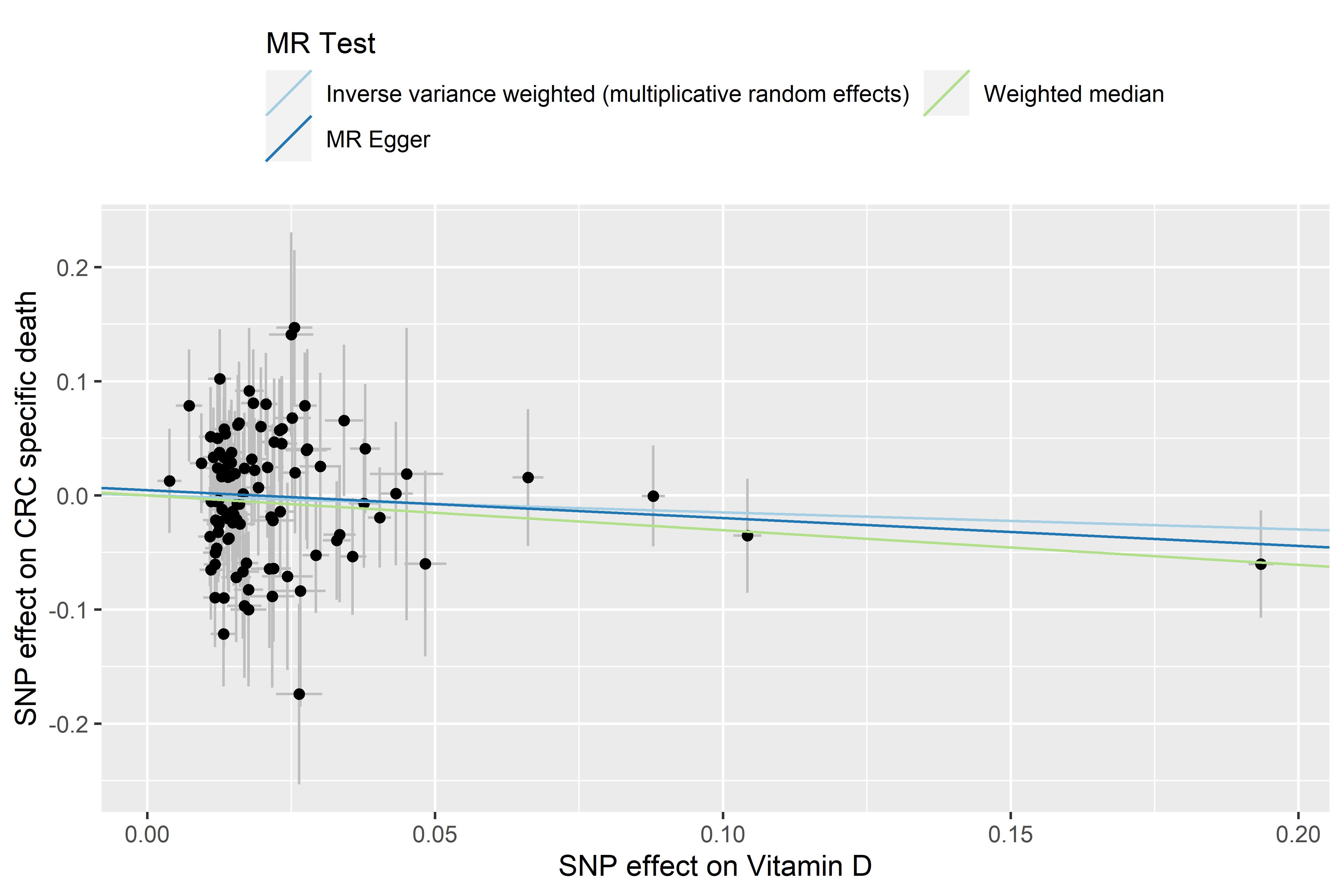

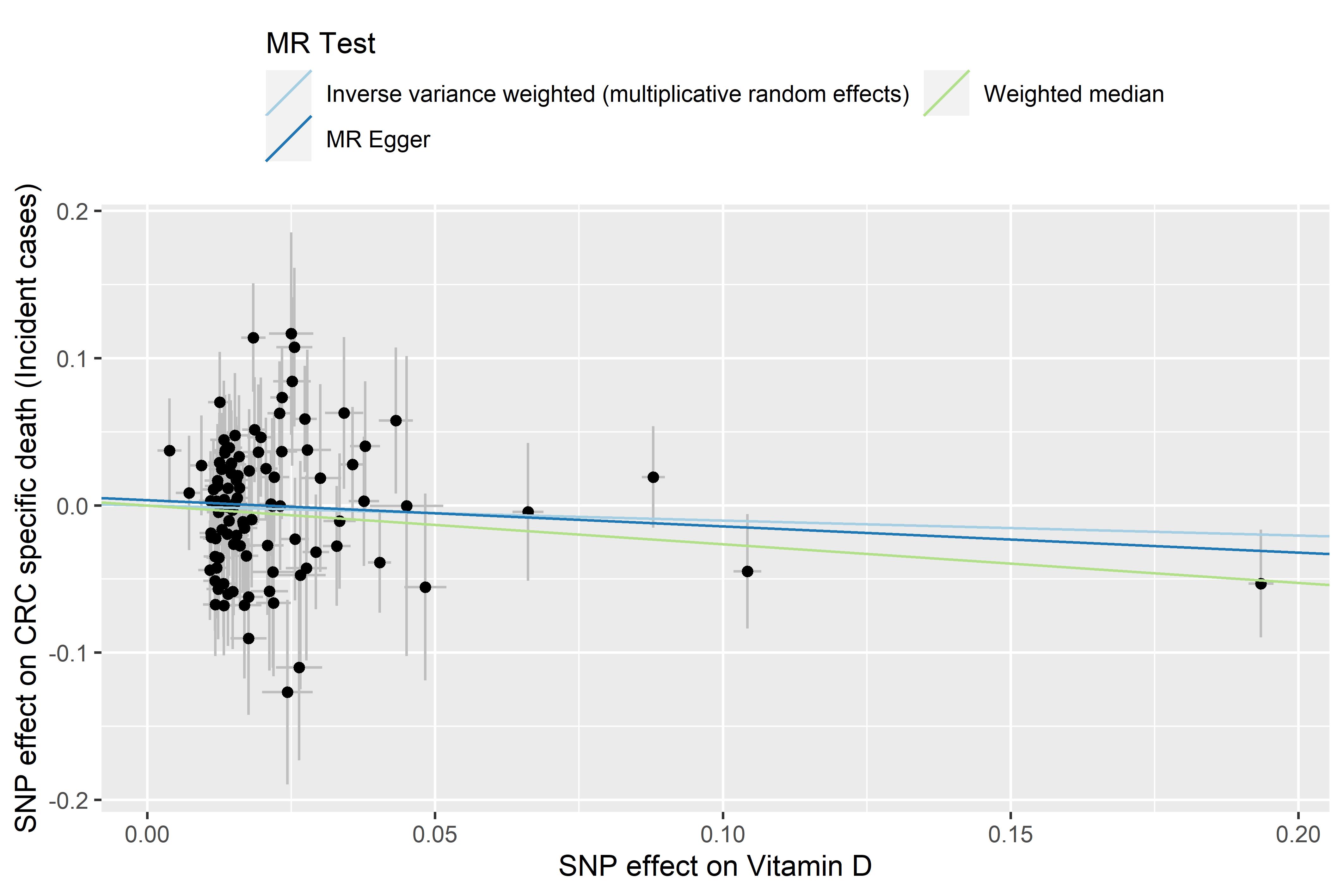


1. Theodoratou E, Kyle J, Cetnarskyj R, Farrington SM, Tenesa A, Barnetson R, et al. Dietary flavonoids and the risk of colorectal cancer. Cancer epidemiology, biomarkers & prevention : a publication of the American Association for Cancer Research, cosponsored by the American Society of Preventive Oncology. 2007;16(4):684-93.

2. Knox S, Harris J, Calton L, Wallace AM. A simple automated solid-phase extraction procedure for measurement of 25-hydroxyvitamin D3 and D2 by liquid chromatography-tandem mass spectrometry. Ann Clin Biochem. 2009;46(Pt 3):226-30.

3. Bycroft C, Freeman C, Petkova D, Band G, Elliott LT, Sharp K, et al. The UK Biobank resource with deep phenotyping and genomic data. Nature. 2018;562(7726):203-9.

4. McCarthy S, Das S, Kretzschmar W, Delaneau O, Wood AR, Teumer A, et al. A reference panel of 64,976 haplotypes for genotype imputation. Nat Genet. 2016;48(10):1279-83.

5. Walter K, Min JL, Huang J, Crooks L, Memari Y, McCarthy S, et al. The UK10K project identifies rare variants in health and disease. Nature. 2015;526(7571):82-90.

6. Dilthey A, Leslie S, Moutsianas L, Shen J, Cox C, Nelson MR, et al. Multi-population classical HLA type imputation. PLoS computational biology. 2013;9(2):e1002877.

7. **Zgaga L**, Theodoratou E, Farrington SM, Din FV, Ooi LY, Glodzik D, et al. Plasma vitamin D concentration influences survival outcome after a diagnosis of colorectal cancer. Journal of Clinical Oncology. 2014;32(23):2430-9.

8. Zgaga L, Theodoratou E, Farrington SM, Agakov F, Tenesa A, Walker M, et al. Diet, environmental factors, and lifestyle underlie the high prevalence of vitamin D deficiency in healthy adults in Scotland, and supplementation reduces the proportion that are severely deficient. J Nutr. 2011;141(8):1535-42.

9. Revez JA, Lin T, Qiao Z, Xue A, Holtz Y, Zhu Z, et al. Genome-wide association study identifies 143 loci associated with 25 hydroxyvitamin D concentration. Nature Communications. 2020;11(1):1647.
